# Supplementary material for: Inclusion bias affects common variant discovery and replication in a health-system linked biobank
Source: Am J Hum Genet. 2026 Mar 10;113(4):702–14. doi: 10.1016/j.ajhg.2026.02.011 (PMC13087405; doi:10.1016/j.ajhg.2026.02.011)
Supplement: Document S1. Figures S1–S6 [file mmc1.pdf]

The American Journal of Human Genetics, Volume *113*

Supplemental information

Inclusion bias affects common variant discovery and replication in a health-system linked biobank

Aditya Pimplaskar, Junqiong Qiu, Sandra Lapinska, Veronica Tozzo, Jeffrey N. Chiang, Bogdan Pasaniuc, and Loes M. Olde Loohuis

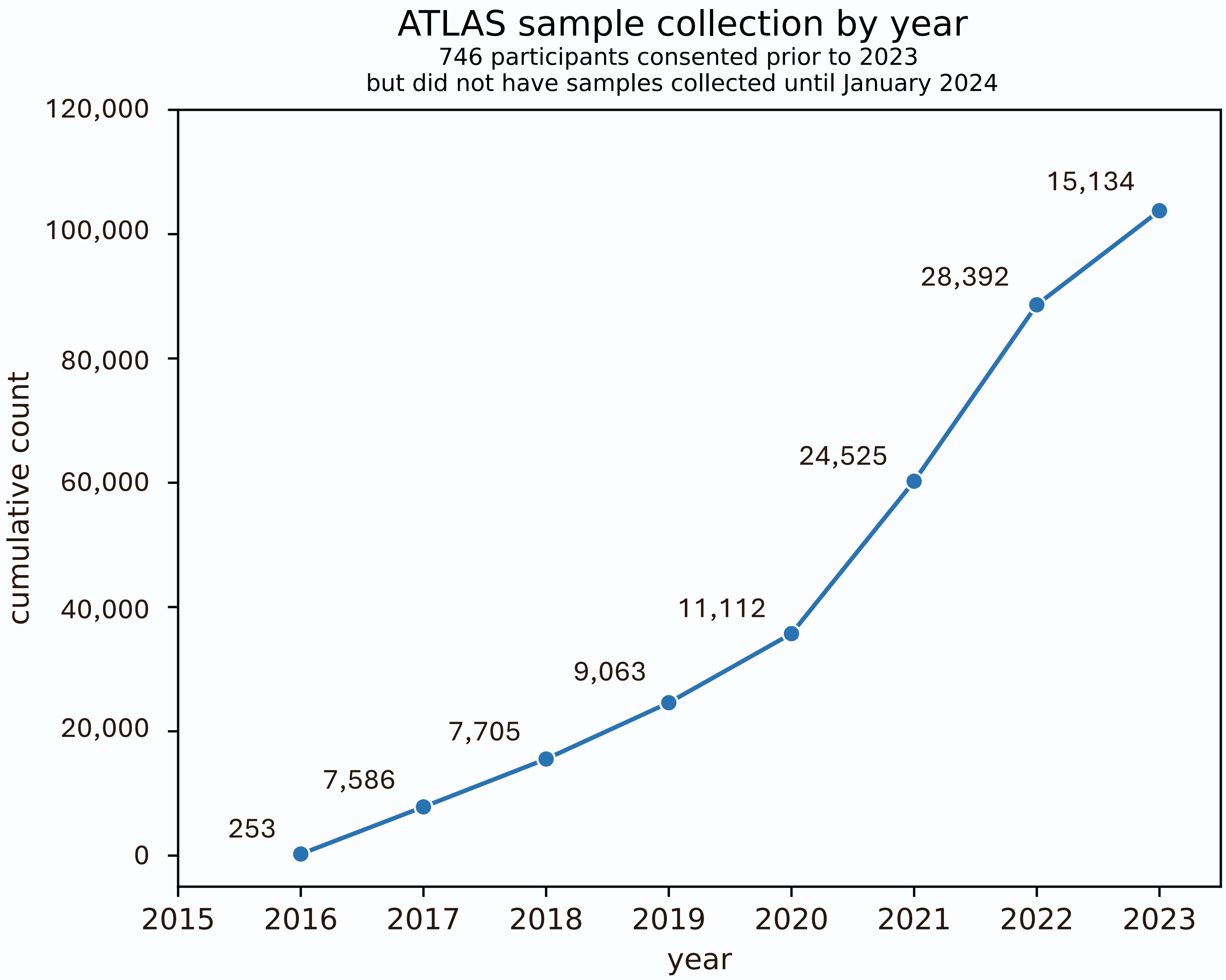

Figure S1. ATLAS sample collection counts by year: Cumulative and yearly counts of ATLAS sample collection by year, spanning enrollment for 2016-2023. A subset of individuals (n=746) individuals who consented into the biobank in 2023 but did not have samples collected until January of 2024 are not included in counts.

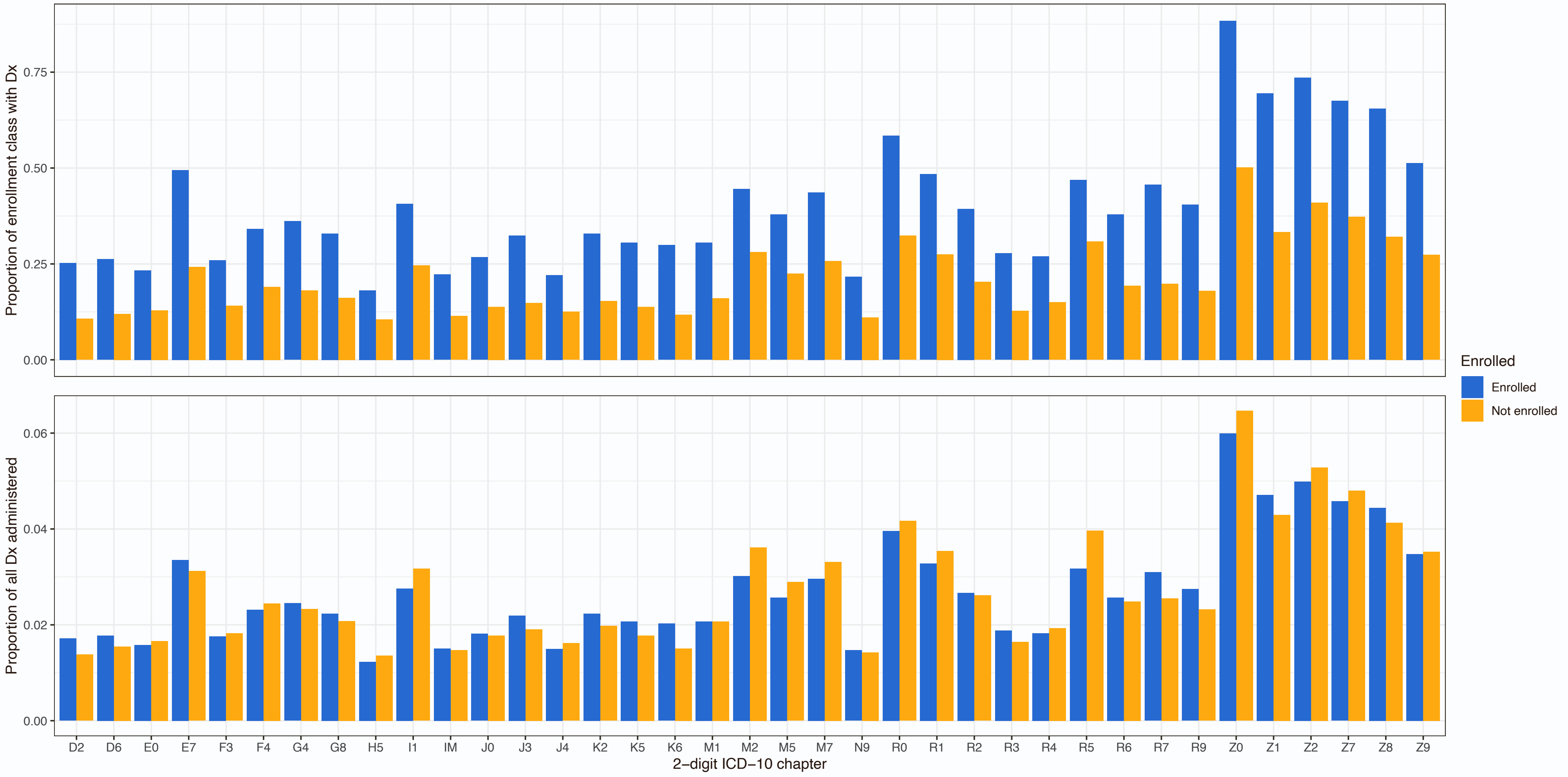

Figure S2. Comparison of diagnostic burden stratified by enrollment : Proportion of individuals stratified by enrollment with a given 2-digit ICD-10 diagnosis (top). Proportion of 2-digit ICD-10 diagnosis relative to all diagnoses administered within enrolled/unenrolled individuals (bottom).

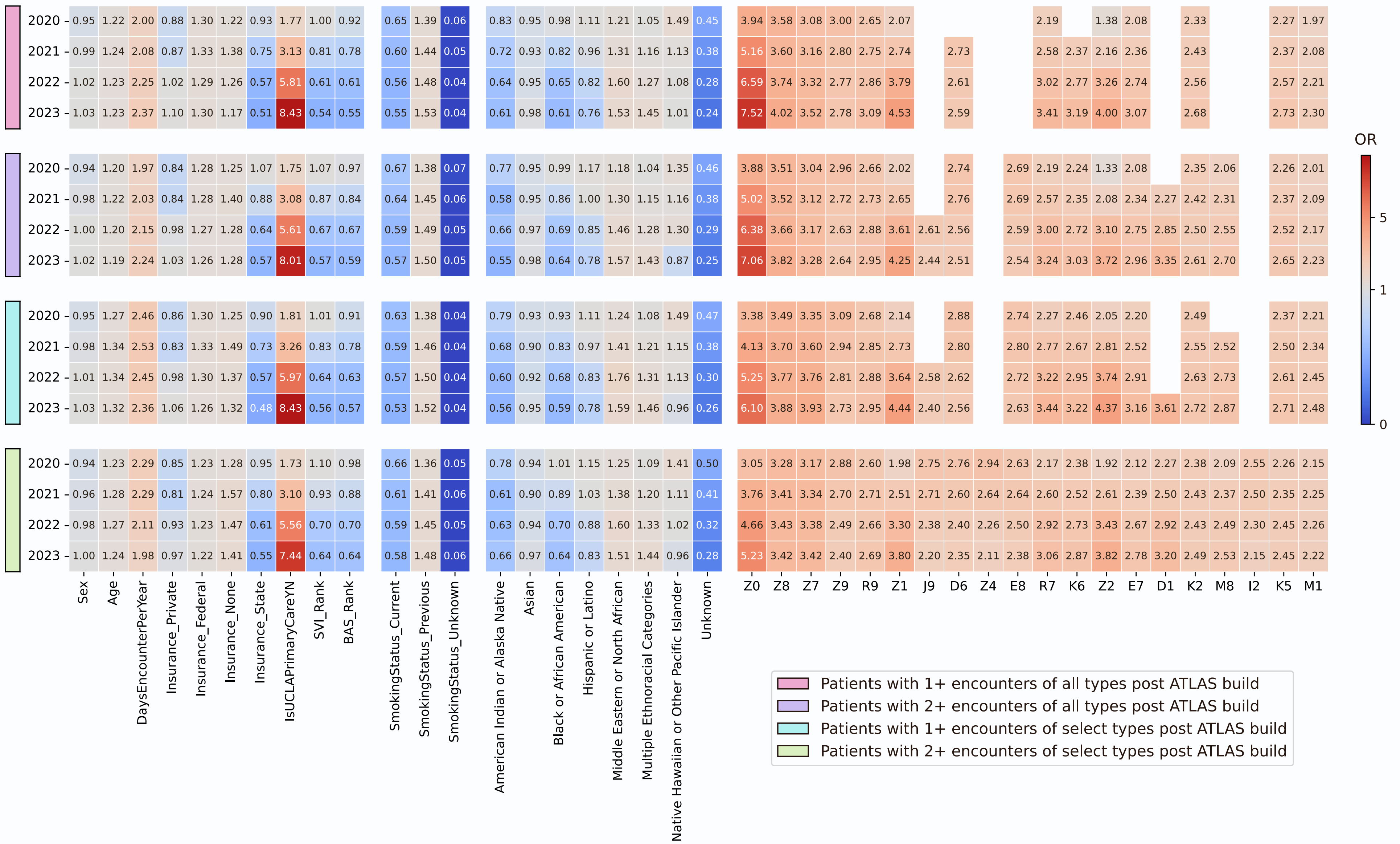

Figure S3. Comparison of feature level effects on enrollment across healthcare utilization inclusion criteria and aggregated across ATLAS recruitment timeframes: Feature level odds ratios compared across data horizons. For a given year, all individuals enrolled by that year were aggregated and compared to a reference consisting of unenrolled individuals up to the data horizon .

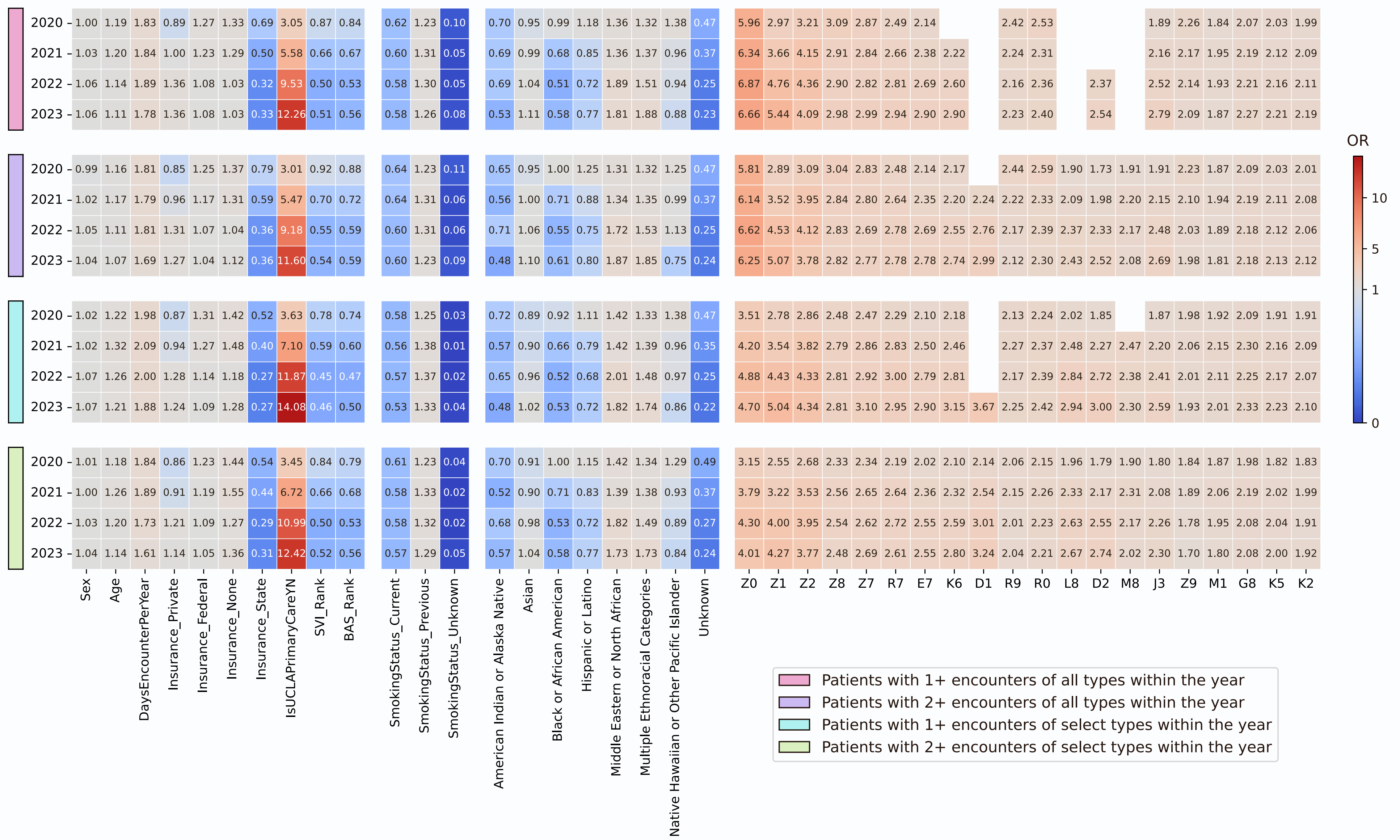

Figure S4. Comparison of per-year feature level effects on enrollment across healthcare utilization inclusion criteria: Feature level odds ratios compared across enrollment years. For a given year, all individuals enrolled by that year were aggregated and compared to a reference consisting of unenrolled individuals up to the data horizon. Individuals who had enrolled in years prior were omitted from the analysis.

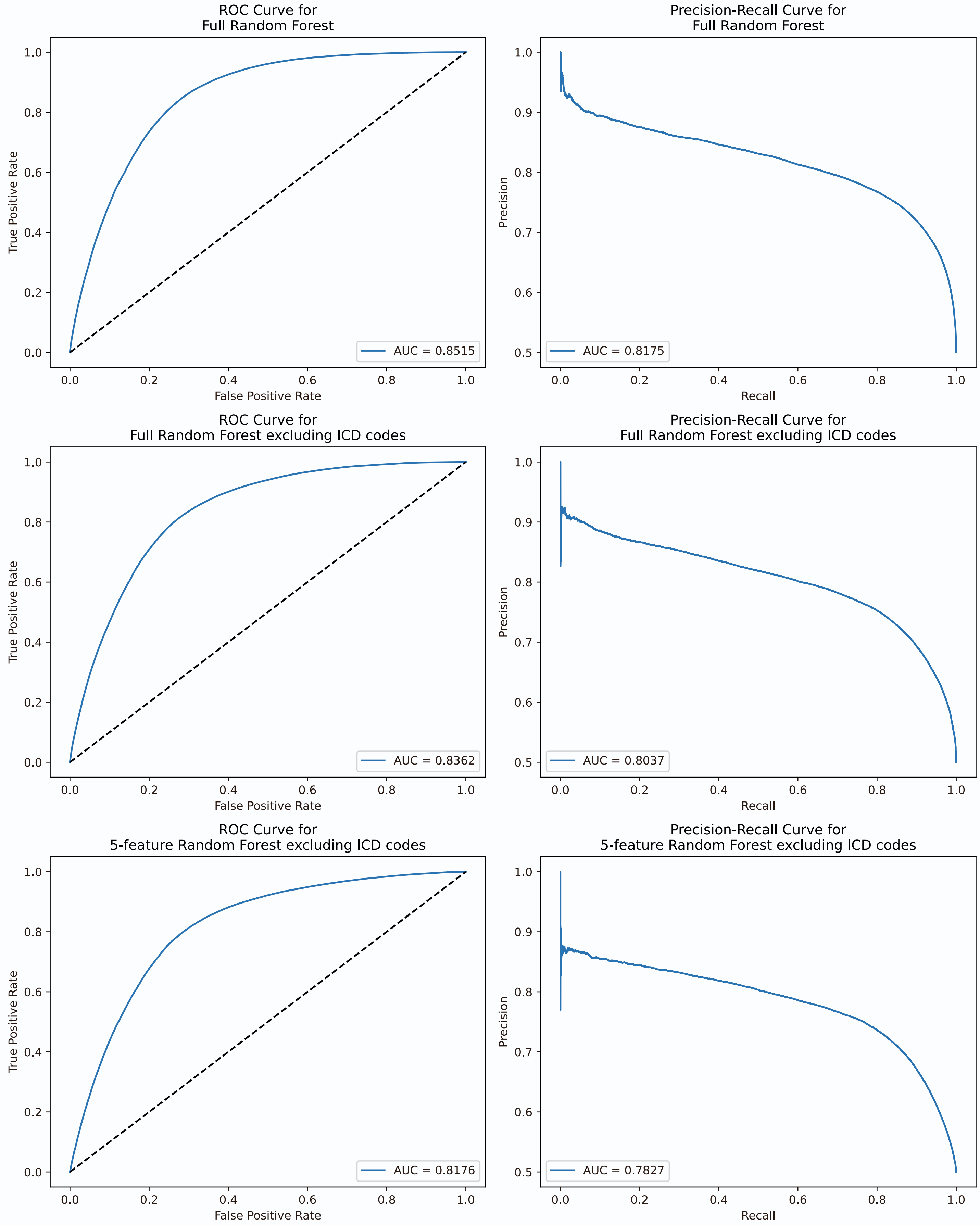

Figure S5. Random forest model performance across model settings: Assessment of random forest model performance using three different model formulations: full model, full model excluding ICD-10 codes, 5-feature recursive feature elimination

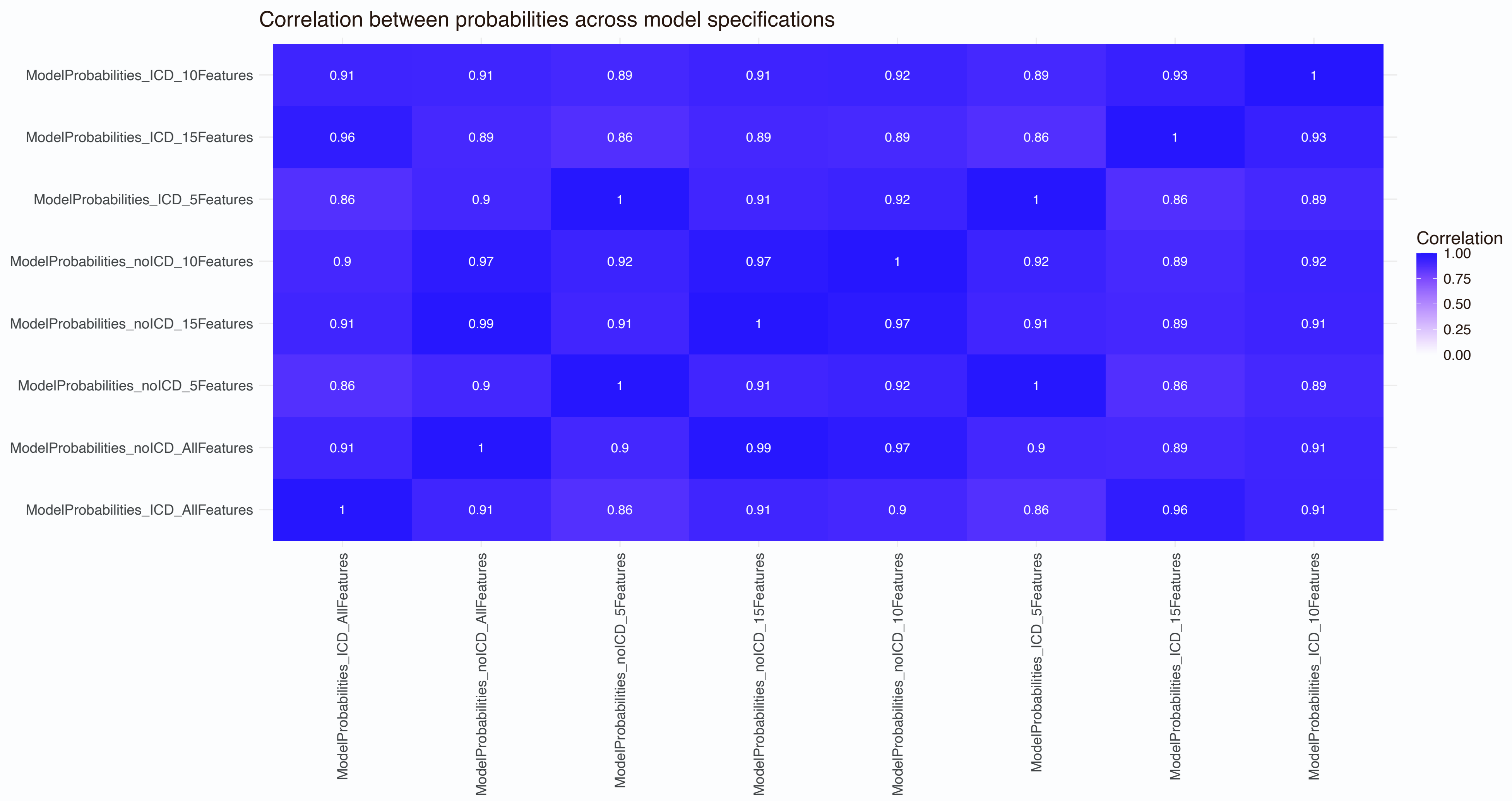

Figure S6. Correlation between enrollment probabilities across model specifications: Pearson correlation of enrollment probability varying recursive feature elimination cutoffs (5, 10, 15 features) and inclusion of ICD-10 codes in feature lists

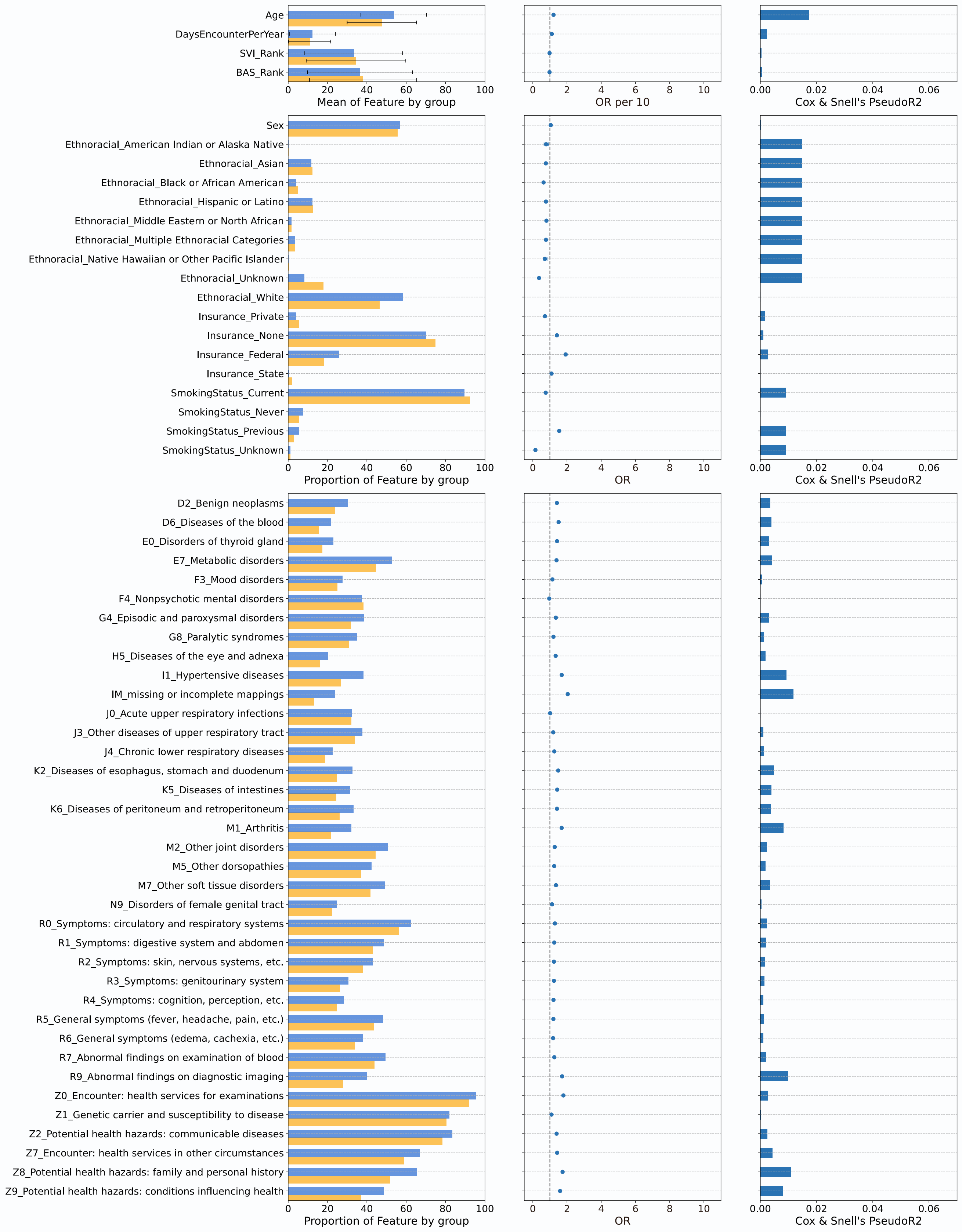

Figure S7. Feature distributions and univariate association statistics for enrollment in a subset of individuals receiving primary care at UCLA: (Left) Feature level distributions stratified by ATLAS enrollment (yellow = not enrolled in ATLAS, blue = enrolled in ATLAS) in a subset of patients receiving primary care at UCLA. For quantitative variables, feature means are depicted, and for categorical features, proportion of individuals in both groups in the feature group are depicted. For diagnostic chapters, any diagnosis in the chapter is sufficient. Reference levels for categorical features are as follows: Sex (male), Ethnoracial category (White), Smoking Status (Never). (Middle/right) Results from univariate associations with ATLAS enrollment in the UCLA Primary Care subset – Odds ratio (middle) and Cox & Snell's pseudo-R2 (right). Effect sizes for quantitative features are presented in the top panel as odds ratios per 10 units.

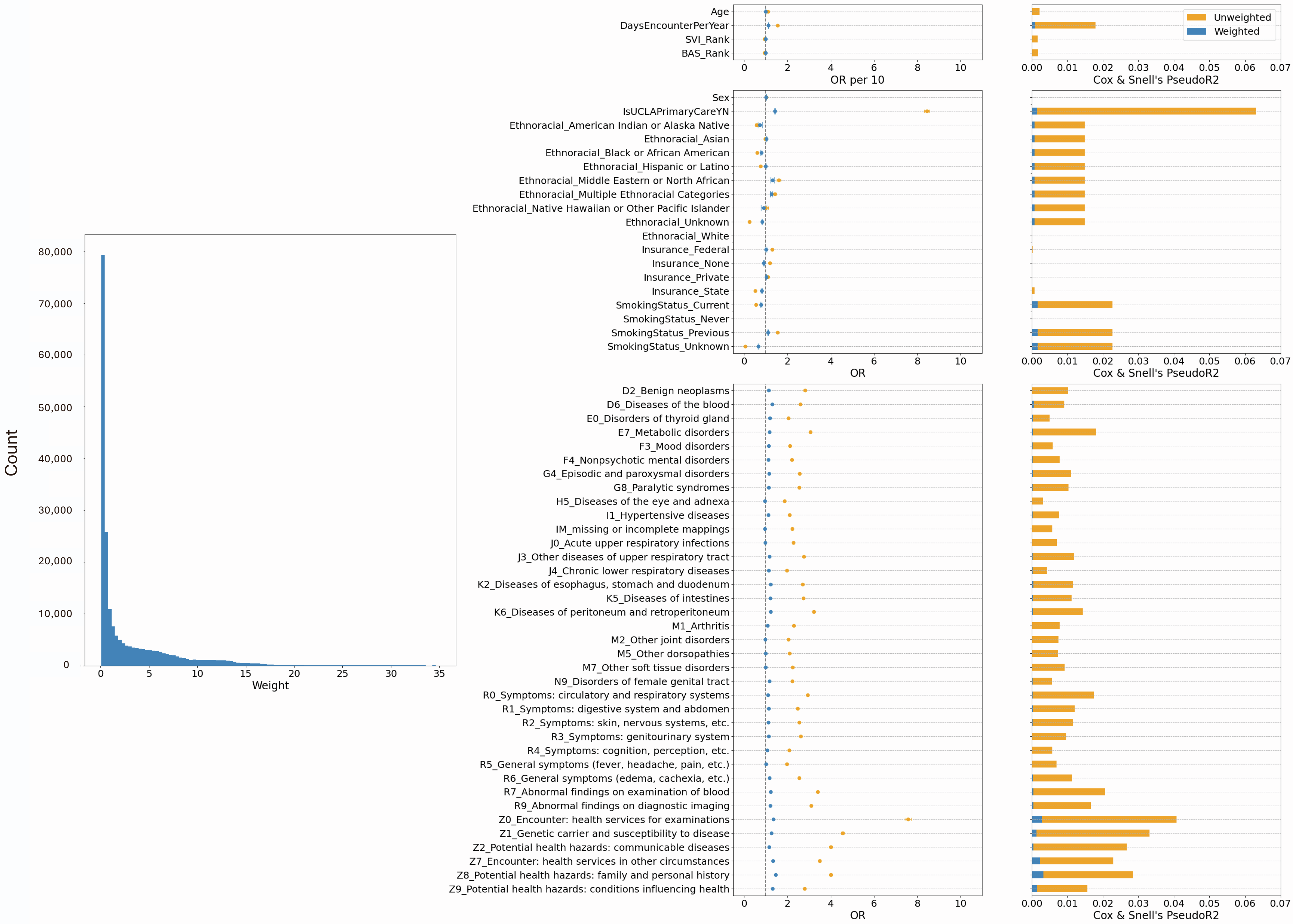

Figure S8. Weight distribution and effect of inverse-probability adjustment on univariate associations: Weights derived from transformation of enrollment probabilities using the 5-feature RFE model (left). Comparison of feature-level univariate effects and variance explained (right) between unweighted associations (blue) and weighted associations (orange).

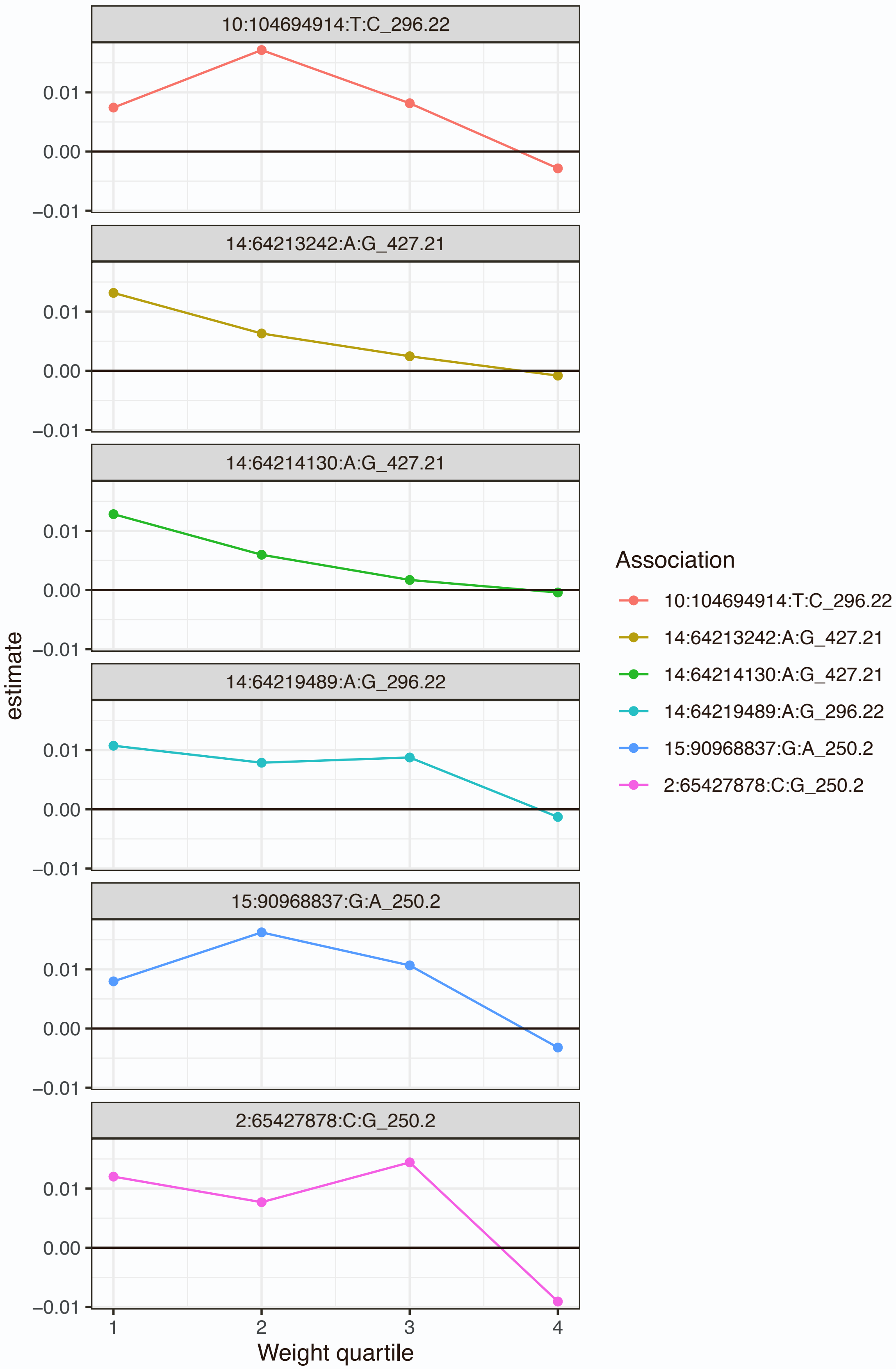

Figure S9. Quartile-stratified effect sizes in associations significant and discordant in the weighted model setting but replicated in the unweighted setting: Unweighted model effect sizes stratified by quartiles of the weight distribution in the 6 associations replicated in the unweighted model setting that were significant with discordant directions of effect in the weighted setting. Model formulations were identical to the aggregate analysis, but run within each individual weight quartile.

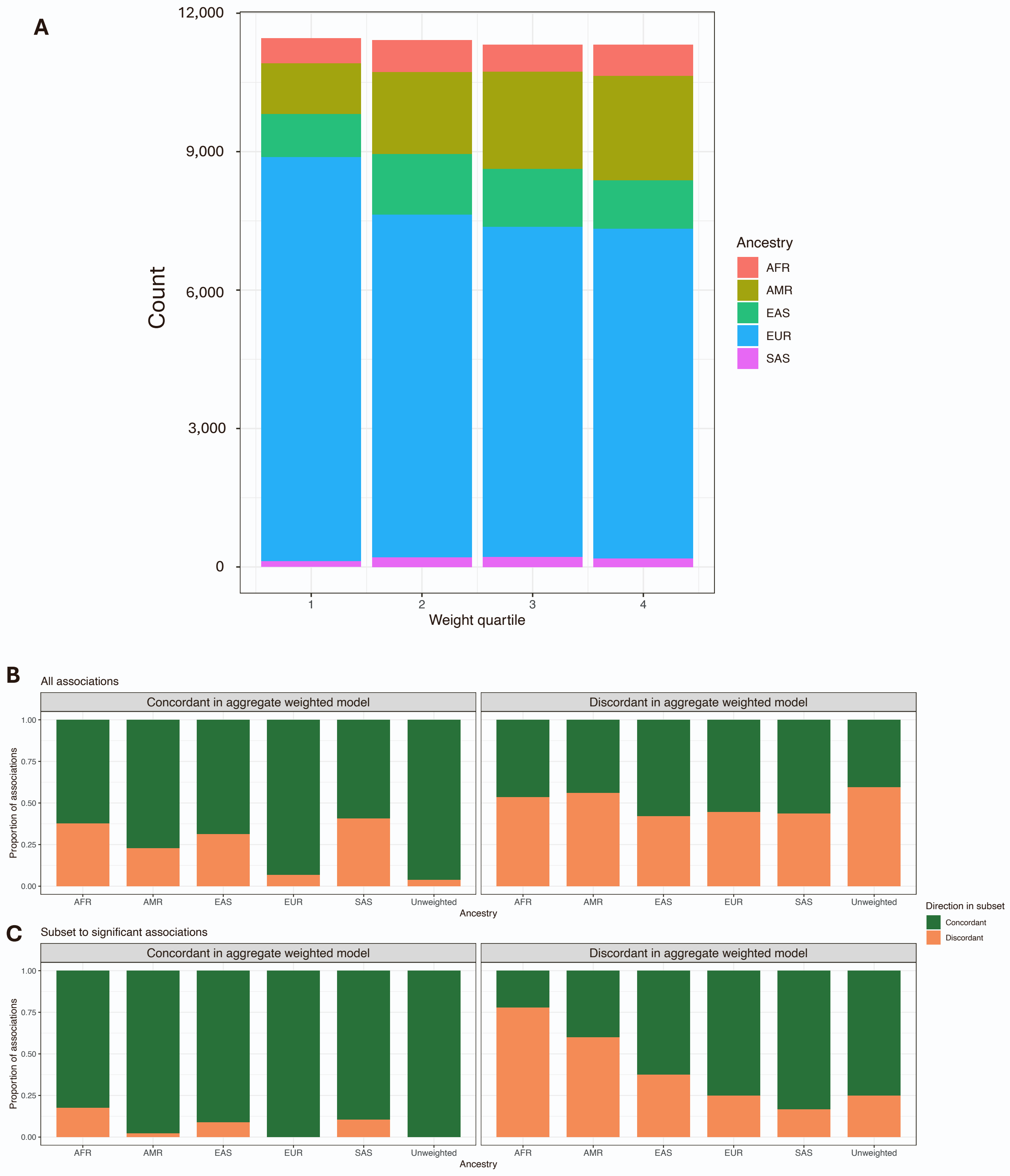

Figure S10. Relationship between genetic ancestry, weight quartile, and concordance of associations: (A) Distribution of genetic ancestry groups across weight quartiles. Within each quartile, individuals for whom an ancestry assignment could not reliably be made were omitted. (B) Proportion of concordant effects of associations in the weighted setting in ancestry-stratified analyses, split by whether the association was significant & concordant (left panel n=724) or significant & discordant (right panel, n=114) compared to pgrm . (C) As in panel B, but restricting associations to those significant in the ancestry-stratified analyses.

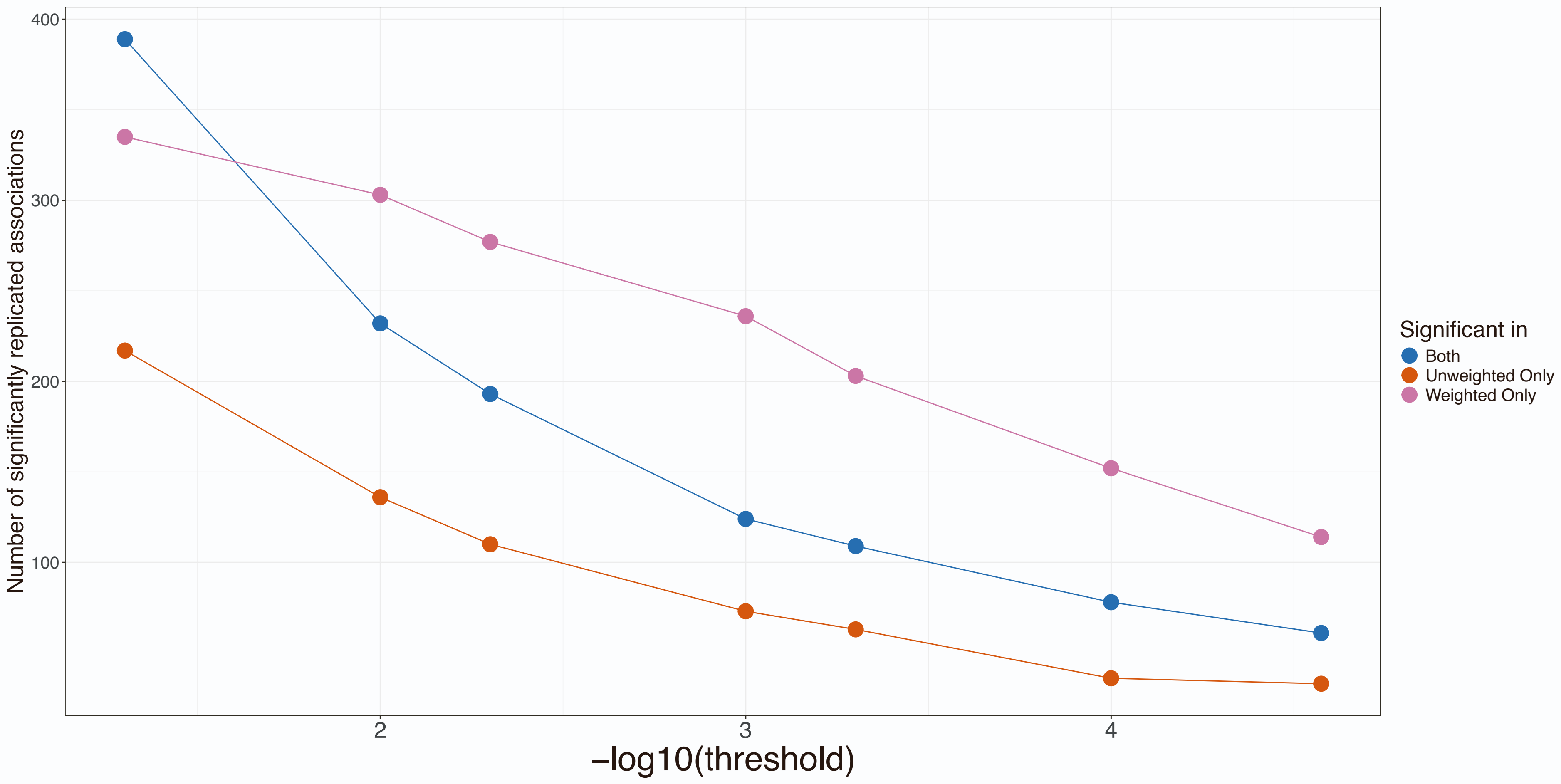

Figure S11. Pgrm variant replication statistics at varying p-value thresholds: Number of significantly replicated associations under each model scheme across p-value thresholds. Replication criteria were p < threshold and consistent direction of effect with pgrm reference.

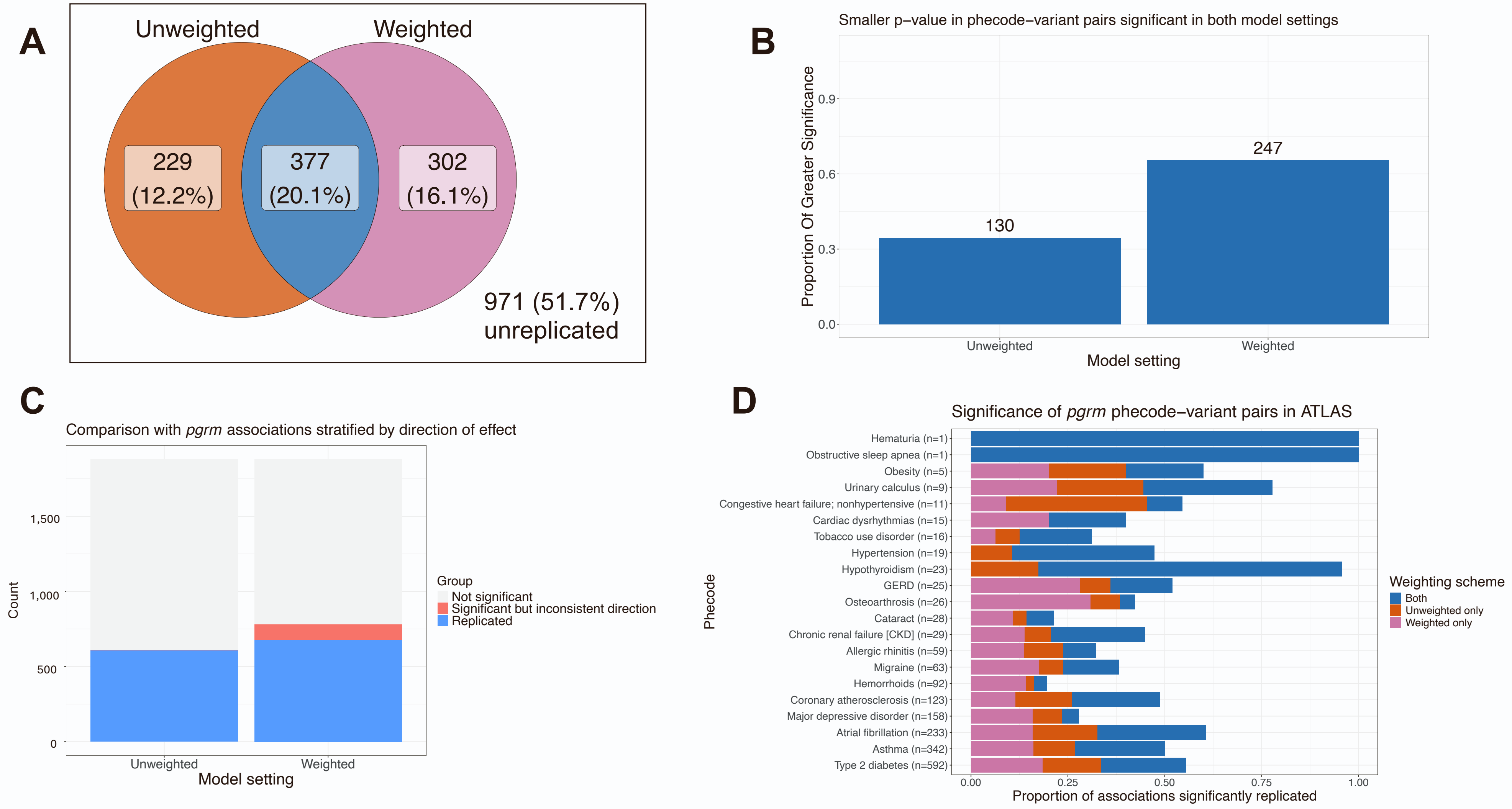

Figure S12. Replication metrics of pgrm variant associations stratified by model setting using the full RF model: Weighted models were adjusted using probabilities from the full random-forest model in place of the recursive-feature eliminated model. A) Venn diagram of counts and percentages of replicated associations from pgrm in the ATLAS sample. Associations were considered replicated at a significance level of $p < 0.05$ and when direction of effect was consistent with pgrm. B) Proportions of associations with smaller p-value stratified by model setting among associations significant under both model settings. C) Proportion of associations with discordant sign with pgrm stratified by model setting. D) Replication across model schemes for variant-phecode associations across 21 phecodes. The number in parentheses indicates the number of queried associations per phecode.

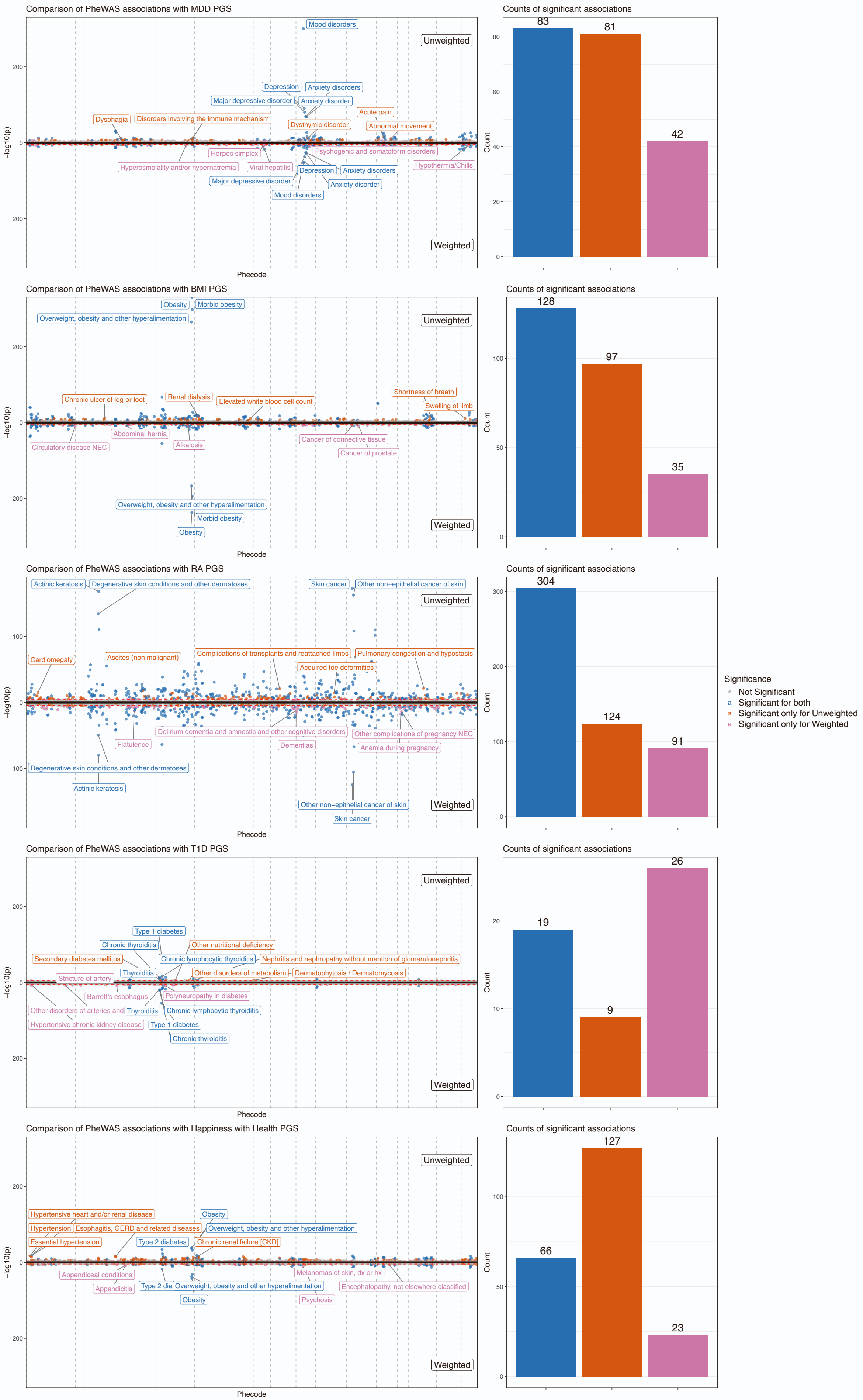

Figure S13. Miami plots of unweighted and weighted PGS-PheWAS associations for 5 PGS: Miami plots for PheWAS on PGS for MDD, BMI, RA, T1D, and Happiness with one's own health (top: unweighted, bottom: weighted) (left). Overall counts of unique and shared associations under model schemes (right)

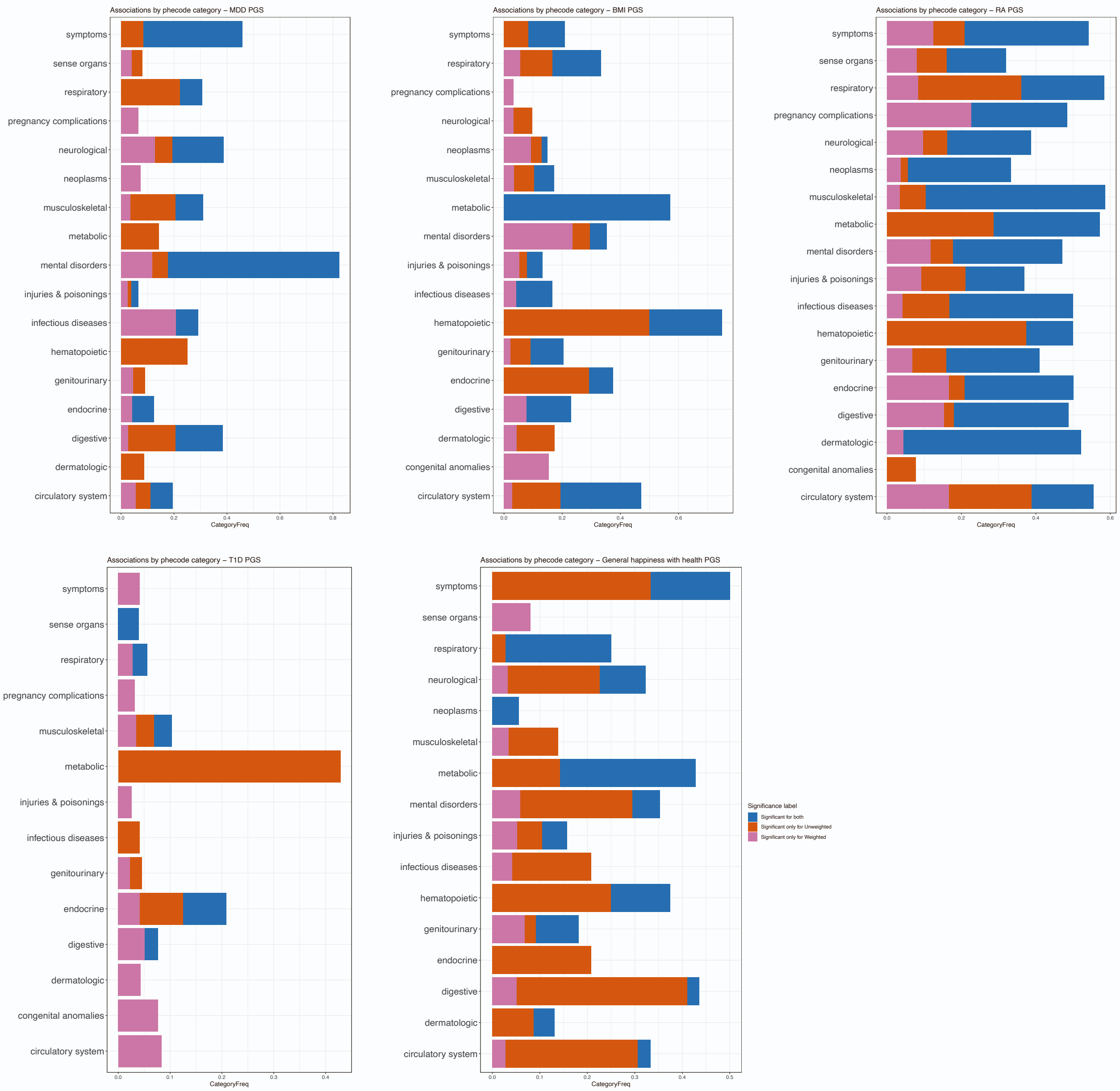

Figure S14. Phecode category breakdown of PheWAS, including associations significant in weighted setting only: Phecode-stratified counts of associations under the unweighted and weighted model schemes, including associations found to be significant only in the weighted setting, for MDD, BMI, RA, T1D, and Happiness with one's own health PGS. Significance was assessed using a Bonferroni-corrected p-value threshold for 1,117 phecodes tested of $p < 4.44e-5$.

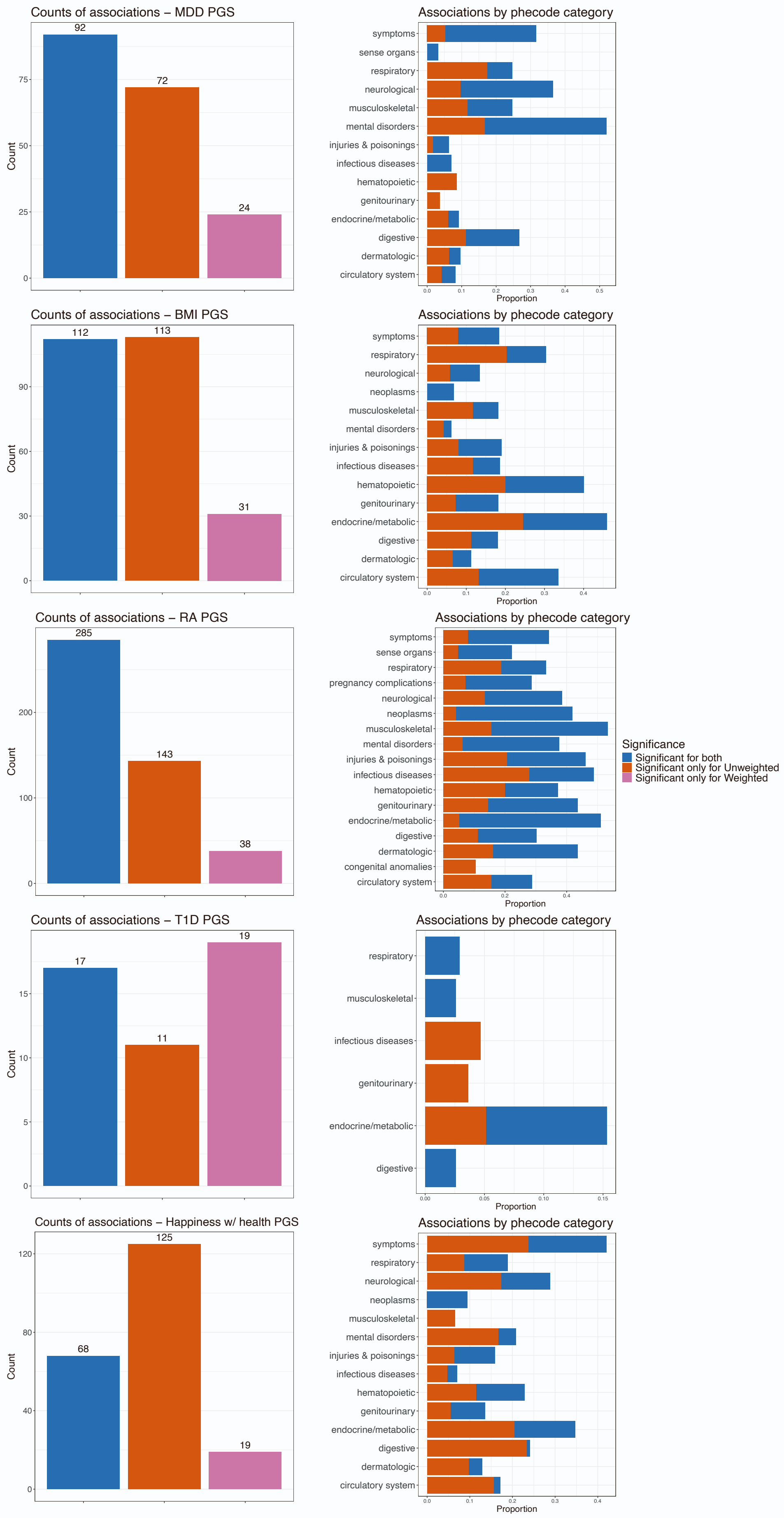

Figure S15. Comparison of unweighted and weighted PGS-PheWAS associations for 5 PGS using the full RF model: Weighted models were adjusted using probabilities from full random-forest model in place of recursive-feature eliminated model. Overall (left) and phecode-stratified (right) counts of shared and unique associations under the unweighted and weighted model schemes for MDD, BMI, RA, T1D, and Happiness with one's own health PGS. Significance was assessed using a Bonferroni-corrected p-value threshold for 1,117 phecodes tested of p < 4.44e-5.

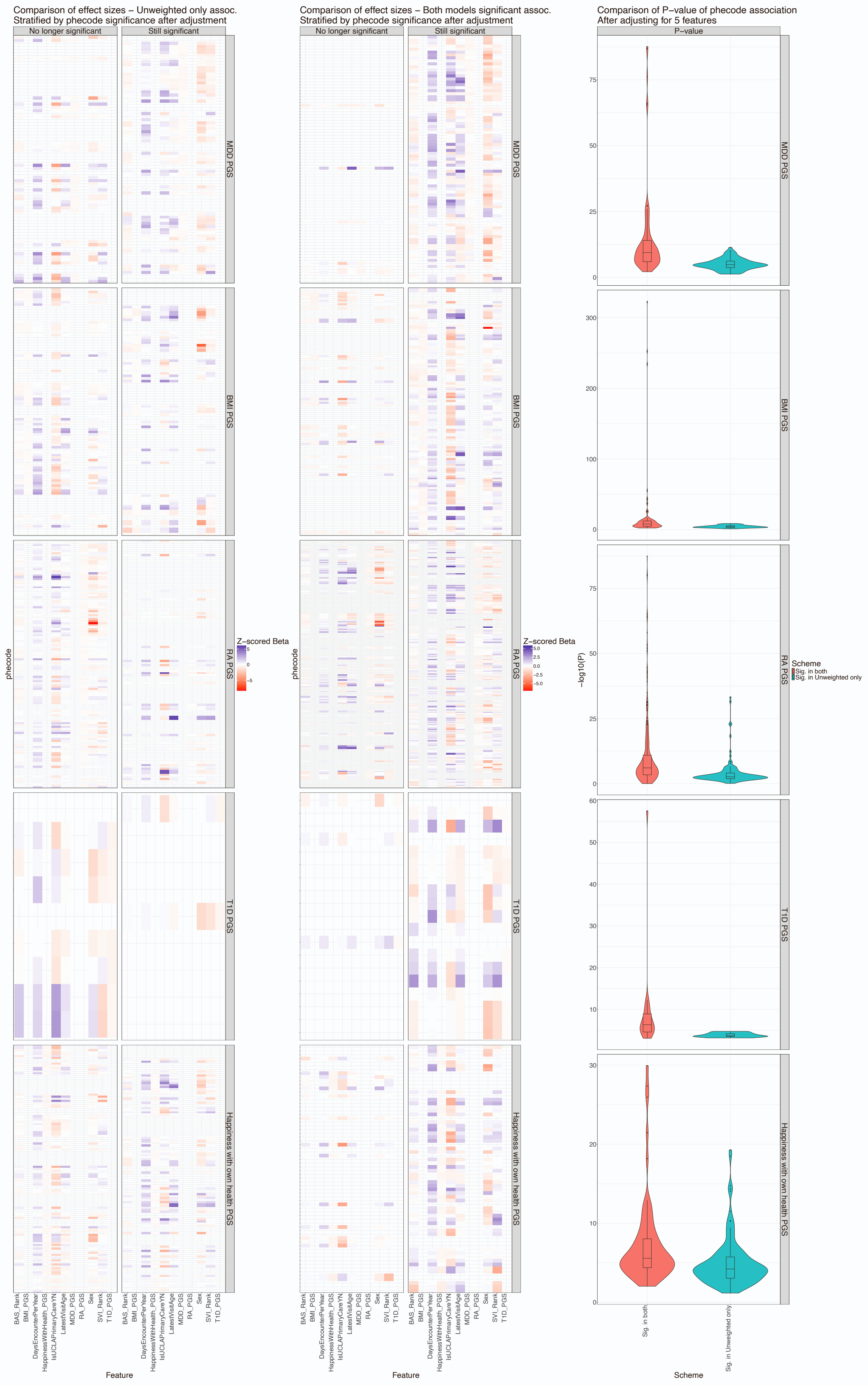

Figure S16. Associations of RF model features following direct adjustment in PGS-PheWAS models: Comparisons of effect sizes (z-scored betas within PGS groups) for RFE random-forest model features after explicit adjustment in PGS-PheAWS models. Comparison in PGS-phecode association settings found to be significant in unweighted models alone (left) and shared by both unweighted and weighted models (middle) stratified by whether phecode-level associations remain significant after accounting for RF model features in PGS-PheWAS models. Comparison of p-values of association with phecode in PGS-PheWAS models accounting for RF model features stratified by phecodes associated with PGS in the unweighted setting alone (red) and in both unweighted and weighted model settings (blue).
