# Supplementary material for: Inclusion bias affects common variant discovery and replication in a health-system linked biobank
Source: Am J Hum Genet. 2026 Mar 10;113(4):702–14. doi: 10.1016/j.ajhg.2026.02.011 (PMC13087405; doi:10.1016/j.ajhg.2026.02.011)
Supplement: Document S2. Article plus supplemental information [file mmc3.pdf]

Inclusion bias affects common variant discovery and replication in a health-system linked biobank

Graphical abstract

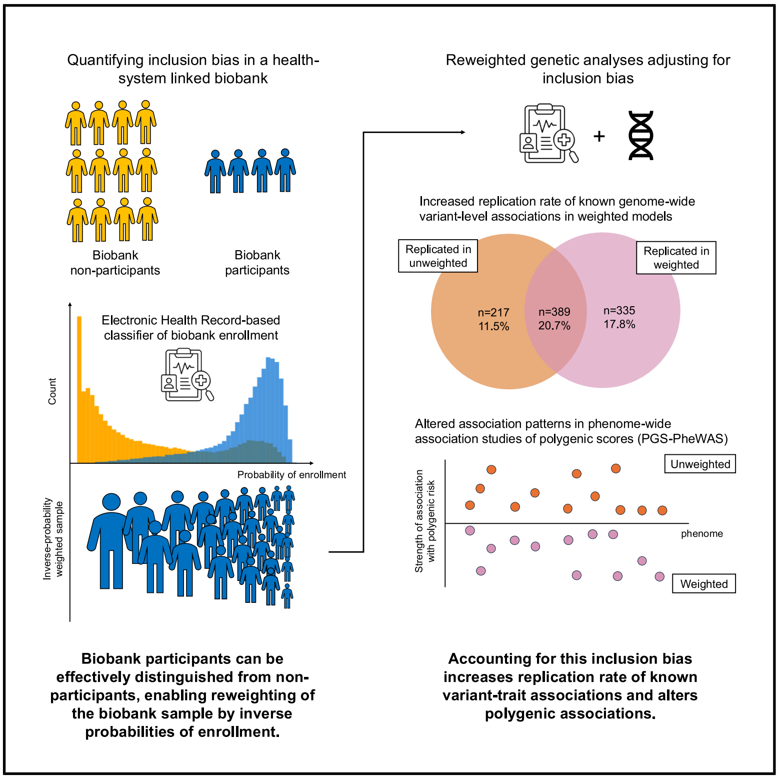

Authors

Aditya Pimplaskar, Junqiong Qiu, Sandra Lapinska, Veronica Tozzo, Jeffrey N. Chiang, Bogdan Pasaniuc, Loes M. Olde Loohuis

Correspondence

loldeloohuis@mednet.ucla.edu

We quantify inclusion bias in a health-system-linked biobank using classification models to distinguish enrolled individuals from the background population. To evaluate its impact on genetic findings observed in biobanks, we reweight analyses by enrollment probability and find increased replication rates of known variant-trait associations and altered polygenic associations.

Pimplaskar et al., 2026, The American Journal of Human Genetics *113*, 702–714
April 2, 2026 © 2026 The Author(s). Published by Elsevier Inc. on behalf of American Society of Human Genetics.
https://doi.org/10.1016/j.ajhg.2026.02.011

Check for updates

CellPress

ARTICLE

Inclusion bias affects common variant discovery and replication in a health-system linked biobank

Aditya Pimplaskar,[1,2,3] Junqiong Qiu,[3] Sandra Lapinska,[4] Veronica Tozzo,[2,3] Jeffrey N. Chiang,[3,5] Bogdan Pasaniuc,[6,7,8] and Loes M. Olde Loohuis[2,3,9,*]

Summary

Electronic health record (EHR)-linked biobanks hold promise for precision medicine by enabling association studies between genetic variants and clinical phenotypes for individual risk assessment. However, most biobanks use opt-in consent protocols to recruit individuals interacting with healthcare systems. This strategy may lead to both participation and recruitment bias, the effects of which on genetic analyses remain understudied. We leverage the UCLA ATLAS Community Health Initiative as a use case to determine possible sources of bias and evaluate their impact on genetic analyses. We find that a wide array of factors are associated with participation, such as receiving primary care at UCLA (odds ratio [OR] = 8.44, $p < 1e{-}300$), frequency of healthcare utilization (OR = 1.04, $p < 1e{-}300$), and various sociodemographic factors. Together, features recorded in EHRs differentiate biobank participants from the broader healthcare system population (area under the receiver operating characteristic curve [AUROC] = 0.85, area under the precision-recall curve [AUPRC] = 0.82). By weighting the sample using inverse probability weights derived from probabilities of enrollment, we replicate 54% more known genome-wide association study (GWAS) variants than models not accounting for bias (e.g., associations between variants in *PPARG* and type 2 diabetes). Potential effects of bias were also present in polygenic score phenome-wide association studies (PGS-PheWAS), where across a panel of five PGS with varying genetic architectures, association patterns were affected by the reweighting strategy. Our results highlight that genetic analyses within EHR-linked biobanks may be affected by participation and recruitment bias and that *ad hoc* analyses within each healthcare system can identify possible sources of confounding.

Introduction

Electronic health record (EHR)-linked biobanks from health systems offer great promise for precision medicine[1–4] and are increasingly common and well powered for the discovery of phenotypic and genetic associations with clinical features.[5,6] These biobanks can facilitate personalized risk assessment by leveraging molecular data to compute polygenic scores (PGSs) as estimates of genetic risk. PGSs can be used to distinguish affected individuals from control subjects,[7–9] subtype into case groups,[10–13] and inform interventions and treatment decisions.[14,15]

Biobanks typically employ opt-in consent protocols and use a variety of recruitment strategies, which can induce biases that, in turn, can impact genetic discovery[16] and clinical utility.[17] Studies comparing participants of the UK Biobank to the background population identified a "healthy participant" bias[18] and demonstrated its effect on the prediction of clinical outcomes, the association of lifestyle and demographic factors with health,[19,20] and estimates of heritability and genetic correlation among traits.[21,22] Different from population-based biobanks such as the UK Biobank, EHR-linked biobanks recruit participants through their interaction with the healthcare system, resulting in participants' increased disease severity and greater healthcare utilization,[23] with a recent study showing that adjusting diagnosis prevalence for recruitment and participation bias impacts PGS prediction for psychiatric conditions.[24] Despite this growing concern, the degree to which inclusion bias affects genetic analyses remains underexplored.

In this study, we analyze an EHR-linked biobank at the University of California, Los Angeles (UCLA), called the UCLA ATLAS Community Health Initiative (ATLAS).[25–27] ATLAS began recruitment in 2016, initially with opt-in protocols in operative and blood testing settings and later using digital messaging through patient portals.[28] The totality of the UCLA EHR spans a variety of care settings and domains and contains health records for over 4 million individuals from the greater UCLA Health System serving the Los Angeles area, 104,516 of whom have enrolled in the ATLAS Initiative. Using ATLAS as a use case, we propose a framework to assess inclusion biases in biobank studies and mitigate their impact on genetic investigations. First, we assess the role of clinical, demographic,

[1]Bioinformatics Interdepartmental Program, UCLA, Los Angeles, CA, USA; [2]Center for Neurobehavioral Genetics, Semel Institute for Neuroscience and Human Behavior, Department of Psychiatry and Biobehavioral Sciences, David Geffen School of Medicine, UCLA, Los Angeles, CA, USA; [3]Department of Computational Medicine, UCLA, Los Angeles, CA, USA; [4]Graduate Group in Genomics and Computational Biology, Perelman School of Medicine, University of Pennsylvania, Philadelphia, PA, USA; [5]Department of Neurosurgery, David Geffen School of Medicine, University of California, Los Angeles, Los Angeles, CA, USA; [6]Department of Genetics, Perelman School of Medicine, University of Pennsylvania, Philadelphia, PA, USA; [7]Department of Biostatistics, Epidemiology and Informatics, Perelman School of Medicine, University of Pennsylvania, Philadelphia, PA, USA; [8]Department of Computer and Information Sciences, School of Engineering and Applied Sciences, University of Pennsylvania, Philadelphia, PA, USA; [9]Department of Human Genetics, UCLA, Los Angeles, CA, USA
*Correspondence: loldeloohuis@mednet.ucla.edu
https://doi.org/10.1016/j.ajhg.2026.02.011.
© 2026 The Author(s). Published by Elsevier Inc. on behalf of American Society of Human Genetics.
This is an open access article under the CC BY license (http://creativecommons.org/licenses/by/4.0/).

and healthcare utilization features on ATLAS enrollment through univariate association analysis. Then, we train a classification model to distinguish the ATLAS-enrolled group from the background population and use its predicted probabilities to generate inverse-probability weights. With these weights, we then test the effects of bias on discovery tasks, including common variant replication and PGS-phenome-wide associations (PGS-PheWASs).

We demonstrate that classification models trained on demographics, healthcare utilization, and diagnostic data can distinguish biobank participants from the background population with high accuracy (area under the receiver operating characteristic curve [AUROC] = 0.85). Incorporating inverse probability weights to account for inclusion bias increased the replication rate of known genome-wide association study (GWAS) associations by 54% and altered the results of PGS-PheWAS scans. Our results suggest that using weighted models to adjust for sources of bias may lead to more robust associations within the biobank.

Subjects and methods

Data description

The UCLA Health Discovery Data Repository (DDR) is a de-identified data repository for UCLA Health System-wide EHRs. The DDR includes data from 2 hospitals and 210 primary/specialty outpatient locations in the UCLA Health System serving Los Angeles County. The UCLA ATLAS Precision Health Biobank includes a diverse sample of individuals from across UCLA Health, whose genomic data can be linked with clinical data from the DDR. Individuals receiving healthcare through UCLA Health were invited to enroll in ATLAS through a universal consent process. “Patient Recruitment and Sample Collection for Precision Health Activities at UCLA” is an approved study by the UCLA institutional review board (UCLA IRB), #17-001013. All participating individuals provided universal consent for the use of residual biological samples. Further details on consent and participation can be found in previous works[26] and at https://www.uclahealth.org/precision-health/programs/atlas-precision-health-biobank. Enrollment was initially offered in inpatient care settings and expanded to other clinical settings, with the latest protocol, as of 2021, allowing enrollment via online patient portals. Participants who provided consent for enrollment in the ATLAS sample allowed residual tissue samples from future clinical visits to be saved for genotyping and further research use.

Cohort querying and characteristics

We considered data from ATLAS participants for which quality-controlled genotypes were available as of December 2024.[26] This subset of individuals was recruited between 2016 and 2023. To analyze the characteristics of the background UCLA Health population, we selected from the DDR those who had at least one encounter between 2016 and 2023, matching the ATLAS recruitment period, and at least one International Classification of Diseases (ICD) code outside of the Z chapter (indicating miscellaneous, non-specific clinical encounters). We further restricted the sample to individuals aged 18–90 between 2016 and 2023, in accordance with the enrollment eligibility criteria of the biobank, and removed individuals with missing self-identified sex.

Feature extraction

We extracted structured EHR features related to demographics, healthcare utilization, and clinical characteristics for the entire UCLA Health population. Demographic features include self-identified sex (male = 0, female = 1), age at the latest recorded visit, self-identified race and ethnicity (ethnoracial category), social vulnerability index (SVI),[29] index for barriers to accessing service (BAS),[30] insurance status, and self-reported smoking status. Individuals for whom sex at birth was missing or was not “male” or “female” were excluded from the cohort. Furthermore, individuals for whom census tract measurements for the SVI and BAS were missing were excluded. Ethnoracial category is a feature defined by UCLA based on self-reported race and ethnicity and includes the values American Indian or Alaska Native, Asian, Black or African American, Hispanic or Latino, Middle Eastern or North African, Native Hawaiian or Pacific Islander, White, multiple ethnoracial categories, and unknown (which included the structured levels for “no usable values” and “unknown” and missing values). The SVI consists of socioeconomic features, household composition, minority status, and housing type, as mapped from the census tract. The BAS utilizes American Community Survey data and aggregates information on citizenship, language, access to broadband, access to health insurance, and vehicles per person over a census tract. Values for the SVI and BAS were recorded on a scale of 1–100, ranked within the UCLA Health System. Insurance status took on the following possible levels: private, state, federal, and none. Smoking status was coded as current smoker, never smoked, previous smoker, or unknown (which included both the structured level for unknown and missing values). Healthcare utilization features included whether individuals received primary care at UCLA, as well as a quantitative feature, DaysEncounterPerYear, that encoded the number of independent visits scaled by the length of an individual’s record in years. For clinical features, we considered the presence in individuals’ records of any 2-digit ICD v.10 (ICD-10) codes with a prevalence greater than 5% (encoded 1 if present and 0 otherwise). ICD-10 codes were considered at any point in time in the records, up to the date of ATLAS enrollment for the enrolled sample and the end of 2023 for non-ATLAS participants.

Univariate analyses

To quantify the influence of individual features on ATLAS enrollment, we used univariate binomial generalized linear models implemented in the *statsmodels* package in Python. Multi-level and categorical features were one-hot encoded. We performed a series of sensitivity analyses to assess the robustness of associations over time and across clinical encounter types. We varied the minimum number of clinical contacts ($\geq$1 or $\geq$2), the types of clinical encounters (all clinical encounters or those specific to the following categories: appointment, office visit, hospital encounter, and lab visit), and the time of recruitment. Since ATLAS recruitment protocols changed over time, we analyze the data by year, for the years 2020–2023, in two ways: we analyzed aggregated enrollment up to a specified year and considered only new enrollment for each year individually. In the latter per-year analyses, we used a sliding window, dropping individuals who had enrolled in previous years and comparing year-specific enrolled individuals

against UCLA Health patients who had not yet enrolled but were eligible to enroll that year. The year 2020 was the first year with a sufficient sample size (>10,000), coinciding with the initiation of the dissemination of universal consent via patient portals. An overview of recruitment numbers by year can be found in Figure S1.

Multivariate random forest models

In order to test our ability to detect enrollment from all available features, we trained random forest (RF) models, allowing us to aggregate individual feature effects to generate reliable enrollment probabilities. Due to the imbalance in sample sizes, we performed 1:1 random downsampling where UCLA Health background population individuals were downsampled to match the number of ATLAS participants. We split our dataset into 10 equally sized folds, ensuring a balanced proportion of ATLAS-enrolled and non-enrolled samples. We trained a model on nine folds and evaluated the model performance on the remaining tenth fold so that each fold was used for evaluation once. Model performance was evaluated using the AUROC and area under the precision-recall curve (AUPRC) metrics. To ensure correct bias adjustment in enrollment probabilities to use in reweighting analysis, we removed clinical features that might be used as outcomes in genetic analysis, and we also performed recursive feature elimination (RFE) to train parsimonious models that only include the most important predictors while preserving model performance. We considered models with 5, 10, and 15 features, respectively, chosen by the RFE based on model-derived rankings of feature importance. Models and RFE feature selection were implemented using the *sklearn*[31] library in Python3. We also evaluated feature importances by computing Shapley values using the TreeExplainer functionality in the *shap*[32,33] package. Unless specified otherwise, the 5-feature model was used for all downstream genetic analyses.

Construction of probability weights

Enrollment probabilities for the ATLAS sample were predicted using both the full random forest model and the smallest 5-feature model following RFE. Probabilities were transformed into weights for downstream models using the following formula[21]:

$$w_i = \frac{(1 - p_i)}{p_i},$$

where w_i is the weight for individual i and their enrollment probability p_i was obtained from the model where individual i was in the held-out evaluation fold. Weights were normalized by dividing by the mean of the overall population distribution, ensuring that the sum of all weights equaled the available sample size. The effective sample size (ESS) due to weighting was computed using a model-agnostic ESS formula that holds across all tests.[34,35]

$$\mathrm{ESS} = \frac{(\Sigma_i {w_i}^2)}{(\Sigma_i w_i)^2}$$

Genetic and phenotypic data collection and preprocessing

Genetic data collection and preprocessing are described in detail elsewhere.[26] In brief, genotyping in ATLAS was performed using a custom genotyping array composed of the Global Screening Array with the multi-disease drop-in panel. The latest release of ATLAS data was aligned to the GRCh38 assembly, with previous releases under GRCh37 lifted over. Genotype data were quality controlled for single-nucleotide polymorphism (SNP) missingness (<5% missing), sample missingness (<5% missing), monomorphic SNPs, and strand ambiguity. Genetic principal components (PCs) were computed using FlashPCA 2.0[36] on genotypes filtered for a minor-allele frequency < 15% and a Hardy-Weinberg p value > 0.001. Genetic ancestry assignments were generated as described elsewhere[25] using the 1000 Genomes[37] reference panel. In brief, ancestries were assigned utilizing a K-nearest neighbors algorithm with superpopulation labels (African [AFR], admixed American [AMR], East Asian [EAS], European [EUR], and South Asian [SAS]) based on clustering along PCs. Individuals for whom genetic ancestry could not be reliably assigned were labeled as "ambiguous" and were excluded from analyses in this work. We retrieved phecodes (v.1.2)[38,39] for all individuals for whom genetic data were available, mapping ICD-10 codes through existing ontologies.[40] Individuals in ATLAS who did not have phecode data were excluded from the following analyses.

Replication of variant-level associations with select phenotypes

The phenotype-genotype reference map *pgrm*[41] catalogs genetic associations spanning over 500 publications for replication studies in biobanks. Using PLINK1.9,[42] we extracted 4,868 SNPs that intersect available ATLAS genotypes and span 5,411 phecode associations from *pgrm*. We filtered phecodes that had low prevalence in ATLAS (<10%), retaining 1,879 associations. The majority of these associations were identified in individuals of EUR genetic ancestry (~78%), followed by EAS (~20%), and <1% from AFR, AMR, and SAS genetic ancestries.

For each association, we assessed the replicability of these associations using unweighted and weighted linear models. In line with previous work,[21,43–45] we chose to apply a linear model since it demonstrates well-defined convergence in weighted regressions. While effect size point estimates are not identical to those of a logistic regression model, since the transformation in logistic regression is a monotonically increasing function of the probability, the direction of effect when distinguishable from zero is always the same. Models were adjusted for self-identified sex and the top 10 genetic PCs to account for population structure and mega-analyzed in the aggregate ATLAS sample. Associations were considered replicated if they had direction consistent with those in the *pgrm* catalog and were significant at a level of $p < 0.05$. No correction for multiple testing was performed, as the objective of this task was not discovery. Two-sample tests for equality of proportions with continuity correction were used to assess the significance of the equality of proportions of replicated associations between model schemes. As a sensitivity analysis, within-ancestry associations were tested separately in each ancestry group using the same weights as in the primary model. Effect sizes were then combined using a fixed-effect inverse-variance-weighted meta-analysis framework, as implemented in the *metafor*[46] package.

Phenome-wide association studies using PGSs

We evaluate the effect of inclusion bias in PGS associations through phenome-wide scans (PGS-PheWAS). For this analysis, all mapped phecodes in the UCLA DDR were used, provided they were present in more than 1% of the available sample and

a phecode category label was available, reducing the 1,839 available phecodes to a set of 1,117. We generated PGSs for five traits that were chosen for their varying complexities of genetic architecture, are frequently diagnosed, and show biological and behavioral associations with a myriad of comorbidities and associated health complications[45,47–50]: major depressive disorder (MDD), body mass index (BMI; PGS ID: PGS000027), rheumatoid arthritis (RA; PGS ID: PGS002088), type 1 diabetes mellitus (T1D; PGS ID: PGS002025), and general happiness with health (PGS ID: PGS002153). Scores for BMI, RA, T1D, and general happiness with health were generated from weights from the PGS catalog,[51,52] while the MDD PGS was generated using the most recent meta-analysis[53] using SBayesR.[54] GWASs used for PGS calculations were performed in samples of predominantly EUR genetic ancestry. We residualized PGSs on the top 10 genetic PCs and calculated Z scores. We performed a phenome-wide scan for each of the five trait PGSs using both unweighted and weighted linear models adjusted by self-identified sex to evaluate their association with phecodes. For each PGS-PheWAS, we applied a Bonferroni correction for the 1,117 phecodes tested at an alpha level of 0.05, resulting in a p value of 4.44e–5.

As a sensitivity analysis, we performed the PGS-PheWAS using weights derived from the full model without RFE. To evaluate the effect of the 5 features used to generate inverse-probability weights, we further ran unweighted PGS-PheWAS associations, explicitly adjusting for these features.

Results

ATLAS and UCLA Health populations have significantly different demographics, diagnostic burdens, and healthcare utilization characteristics

As of December 2024, the UCLA Health System EHR contained records for 1,568,927 individuals receiving care during the recruitment period and meeting our inclusion criteria for analyses. From these, 104,516 individuals consented to participate in the ATLAS Initiative and comprised the enrolled group, with the remainder of the individuals labeled as unenrolled. Individual-level data on who was offered enrollment (and their responses) were unavailable; it is important to note that while ATLAS-enrolled individuals comprised 6.6% of our sample in aggregate, 64% of individuals who were offered enrollment consented to participate. Among our enrolled sample, 54,770 individuals had quality-controlled genotypes available at the time of analysis; of these, 48,664 individuals who also had available phecode data were considered for downstream genetic analyses. We observed significant differences in demographics and healthcare utilization characteristics between the two groups (Figure 1; Table S1). For example, enrolled individuals were more likely to self-report as White/non-Hispanic (57.3%) compared to unenrolled individuals (43%, odds ratio [OR] = 0.604, 95% confidence interval [CI] = [0.58–0.62], p = 1.37e−210 for African American and 0.76 [0.74–0.77], p = 2.24e−180 for Hispanic/Latino). The most striking difference between enrolled and unenrolled individuals is the likelihood of receiving primary care at UCLA: 70.2% of enrolled individuals compared to 21.8% in the unenrolled sample (O.R. 8.44 [8.33–8.56], p < 1e−300). Previous smokers were overrepresented in the enrolled sample (26.4% compared to 15.3%, OR = 1.54 [1.52–1.57], p < 1e−300), while current smokers were underrepresented (3.97% compared to 6.4%, OR = 0.55 [0.53–0.57], p = 1.77e−286). While most individuals use private health insurance at UCLA, enrolled individuals were found to be less likely to use state insurance (2.08%) compared to unenrolled individuals (3.98%, OR = 0.51 [0.49–0.53], p = 6.72e−202).

Across all ICD chapters, we found a significantly higher proportion of individuals with diagnoses in the enrolled sample compared to the unenrolled sample, reflecting a global increase in diagnostic burden (Figures 1 and S2; Table S1). This was also reflected in the overall increased number of visits per year: 12.8 in the enrolled group versus 6.7 in the unenrolled group (OR = 1.54 per increase of 10 days [1.53–1.55], p < 1e−300). Enrolled individuals had enrichment for codes from the R and Z ICD-10 chapters; these are not disease specific but rather encompass symptoms and signs, social determinants of health, and contact with healthcare services,[55] emphasizing the increased healthcare utilization by individuals in the ATLAS cohort. In line with results on healthcare utilization, we found that ATLAS participants were more likely to receive a Z0 ICD-10 diagnosis (“encounter for general examination without complaint, suspected or reported diagnosis”) with an OR of 7.57 [7.43–7.72] (p < 1e-300). Note that the Z0 chapter includes diagnostic codes for routine blood draws, from which residual blood is often used for genotyping. These results were consistent in sensitivity analysis, where cohorts were selected based on a range of healthcare utilization inclusion criteria and across ATLAS recruitment time frames (Figures S3 and S4). Descriptions of 2-digit ICD chapters are provided in Table S2.

A multivariate random forest model can identify enrolled individuals with high accuracy

A random forest model trained on all available demographics, healthcare utilization, and ICD 10 diagnostic chapters was able to discriminate between enrolled and not enrolled individuals with high accuracy, with a mean (SE) 10-fold cross-validation AUROC of 0.85 (0.0025) and AUPRC of 0.82 (0.003) (Figure S5). We assessed feature importances with Shapley values, quantifying feature contributions to model predictions. Results were consistent with univariate associations and included receiving primary care at UCLA Health, the frequency of healthcare utilization, and several ICD-10 chapters of abnormalities in imaging, blood testing, and metabolic disorders (Figure 2A). Furthermore, unknown values for self-report survey-based fields such as ethnoracial category and smoking status were more frequent for unenrolled individuals and were important predictors of enrollment. Models developed through RFE (RFE with 5, 10, and 15

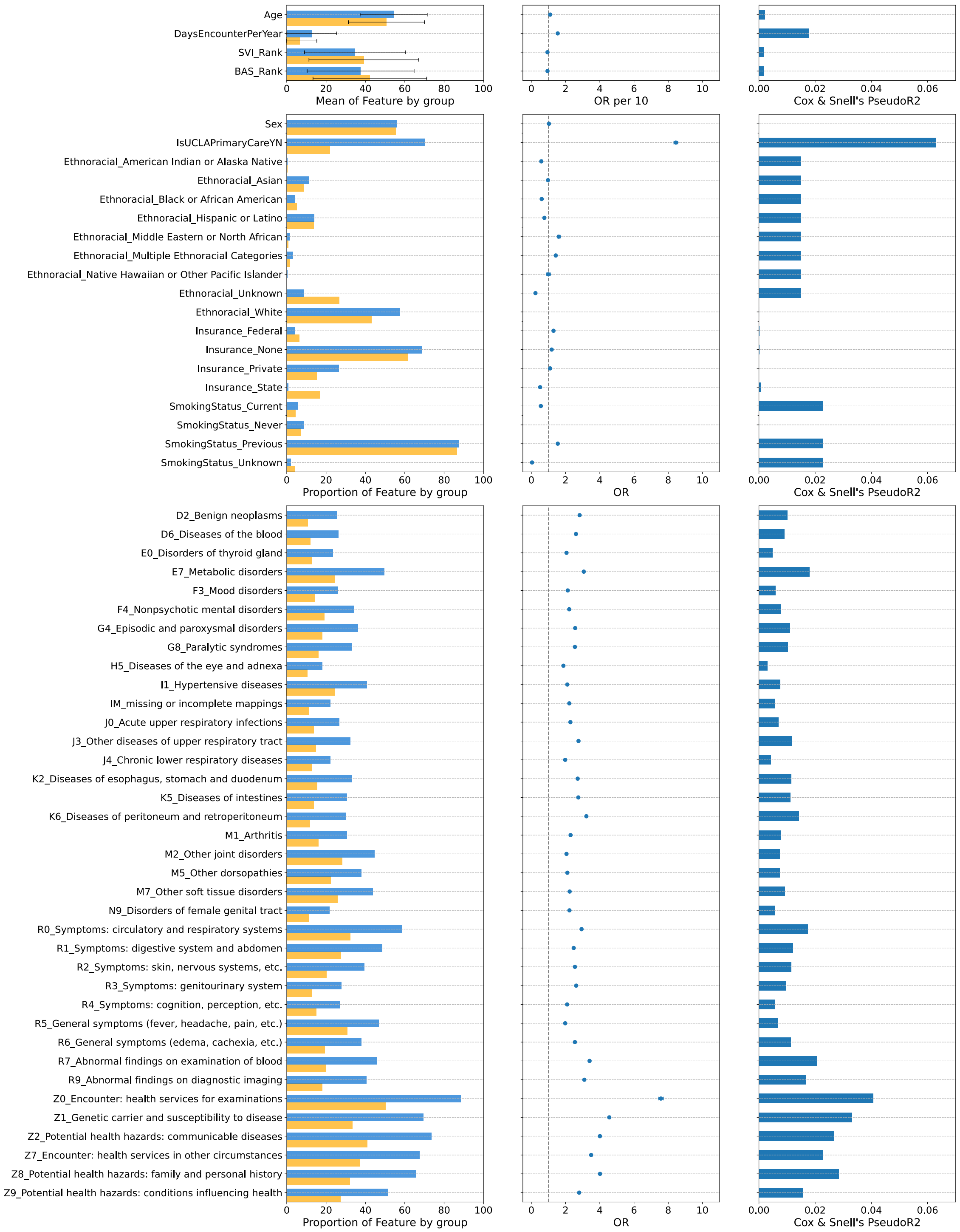

Figure 1. Cohort characteristics of the UCLA Health sample and ATLAS subsample
(Left) Feature-level distributions stratified by ATLAS enrollment (yellow: not enrolled in ATLAS, blue: enrolled in ATLAS). For quantitative variables, feature means are depicted, and for categorical features, the proportion of individuals in both groups in the feature group is depicted. For diagnostic chapters, any diagnosis in the chapter is sufficient. Reference levels for categorical features are as follows: sex (male), ethnoracial category (White), and smoking status (never). (Middle/right) Results from univariate associations with ATLAS enrollment— odds ratio (middle) and Cox and Snell's pseudo-R2 (right), which provides an estimate of variance explained by a given feature based on the likelihood ratio. Effect sizes for quantitative features are presented in the top image as odds ratios per 10 units.

features) still identified ATLAS-enrolled individuals with high accuracy and yielded enrollment probability estimates that were well correlated with the full model (Figures S5 and S6; Table S3). A 5-feature model using age at latest visit, primary care status, frequency of healthcare encounters per year, and indices for SVI and BAS as

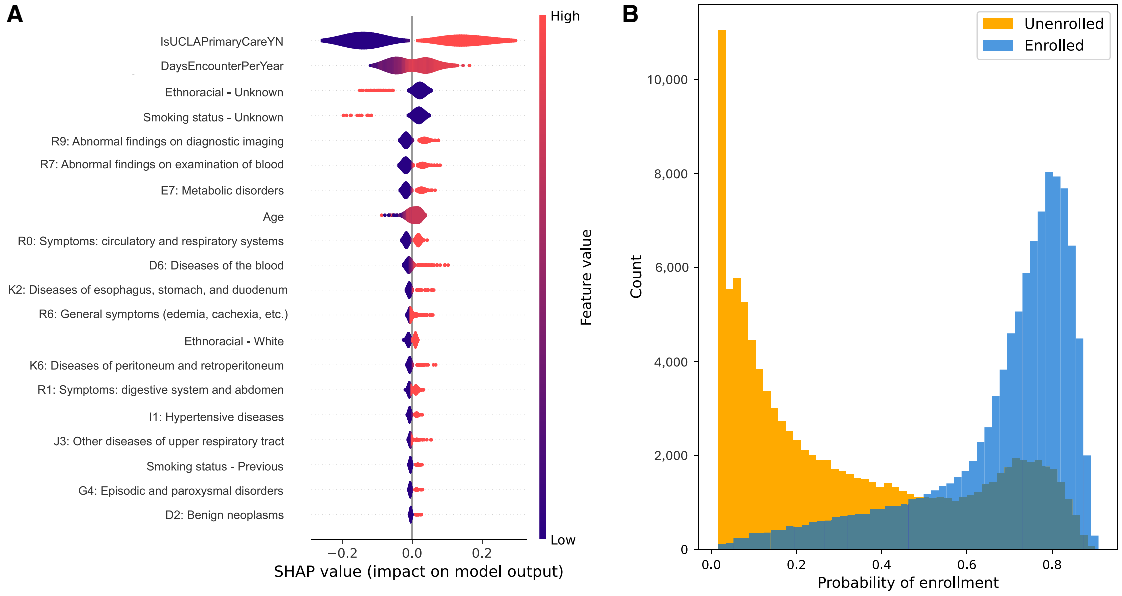

Figure 2. Feature characteristics and probability distributions stratified by enrollment from a multivariate random forest model classifier of ATLAS enrollment

(A) Beeswarm plot of Shapley values on a subset of 100 individuals reveals healthcare utilization patterns and select ICD-10 diagnoses as important predictors of ATLAS enrollment. Descriptions of ICD-10 diagnosis codes can be found in Table S2.

(B) Predicted probability distributions stratified by true ATLAS enrollment status (yellow: not enrolled in ATLAS, blue: enrolled in ATLAS) show strong separation between the classes.

predictors distinguished ATLAS-enrolled from unenrolled individuals with a performance very similar to the full model (AUROC = 0.82 [0.0031], AUPRC = 0.78 [0.004]; Figure S5; Table S3). Since receiving primary care at UCLA was the strongest predictor in our model, we performed a sensitivity analysis to evaluate classification models that included only the subset of primary care patients (n = 400,532), of whom 74,266 were enrolled in ATLAS and 326,266 were unenrolled. While overall classification performance was reduced (AUROC = 0.68 [0.0086], AUPRC = 0.66 [0.002]), univariate associations were in line with the larger model (Table S4; Figure S7).

Inverse probability reweighting attenuates effect sizes in the majority of considered univariate associations with enrollment

We used the multivariate RF model to recover ATLAS-enrollment probabilities. While the majority of enrolled individuals had markedly higher predicted probabilities of enrollment, the distributions of predicted probabilities overlap, with some enrolled individuals having been assigned low probabilities of enrollment, and vice versa (Figure 2B). Enrollment probabilities were transformed and normalized to generate inverse-probability weights. We then tested whether adjusting by reweighting effectively removed the previously observed associations between univariate features and enrollment and observed a dramatic reduction in effect sizes as well as variance explained (Figure S8). Predicted probabilities of enrollment were also recovered from the 5-feature random forest model, and we verified that estimates of enrollment probabilities were well correlated between various model formulations, with a Pearson correlation of 0.86 between the 5-feature model-derived probabilities and the full model-derived probabilities (Figure S6). These enrollment probabilities were transformed and normalized to generate inverse-probability weights, with a mean of 1 (SE = 1.81) and ranging from 0.07 to 25.3. The weights were then used to create a weighted enrolled sample more representative of the background UCLA Health population while at the same time ensuring that no features (such as ICD-10 codes) that were used in the enrollment-prediction models would be used as outcomes in our genetic analyses. In our study data, 48,664 enrolled ATLAS individuals had genetic data available; after reweighting, our ESS reduced to 11,319.9, corresponding to a 4.3-fold reduction.

Replicability of known GWAS variants is improved under a weighting scheme

We tested whether accounting for inclusion biases changed replication of known common trait-variant associations obtained from *pgrm*.[41] Out of the 5,411 SNP-phecode associations in *pgrm*, 1,879 (34.7%) met filtering criteria based on data availability in ATLAS and were tested for association (Table S5). These associations span 27 phecodes, with the majority coming from phecodes 250.2 (type 2 diabetes), 495 (asthma), and 427.21 (atrial fibrillation). Out of all 1,879 associations tested, 20.7% was replicated in both unweighted and weighted settings, and an additional 11.5% was replicated only in the unweighted analysis, while an additional 17.8% was replicated only in the

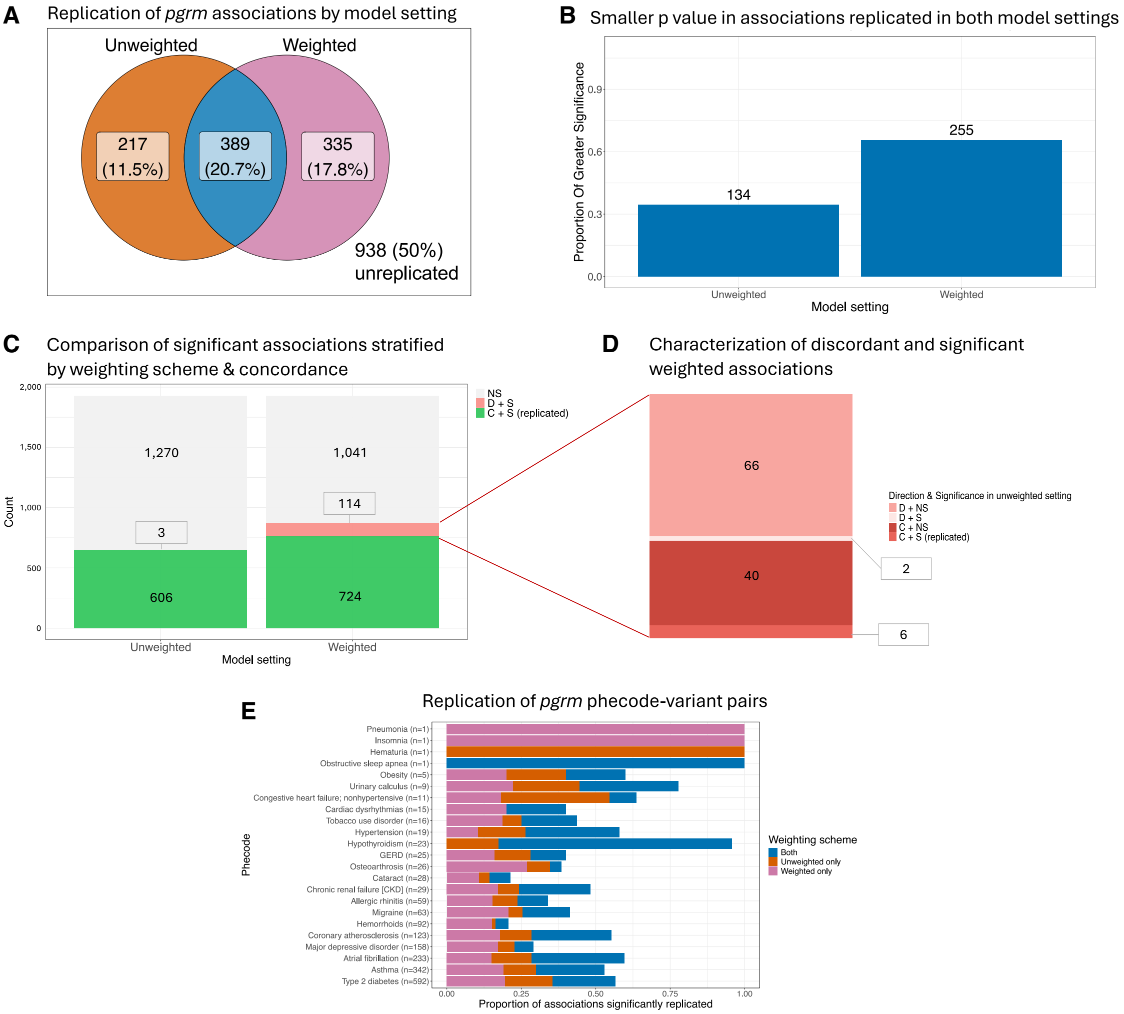

Figure 3. Replication metrics of pgrm variant associations in ATLAS stratified by model setting

(A) Venn diagram of counts and percentages of replicated associations from pgrm in the ATLAS sample. Associations were considered replicated at a significance level of $p < 0.05$ and when the direction of effect was consistent with pgrm.

(B) Proportions of associations with smaller p values stratified by model setting among associations significant under both model settings.

(C and D) Counts of detected associations stratified by model setting (C) and breakdown of significant weighted associations with discordant direction of effect compared to pgrm (D), labeled with respect to their direction of effect and significance in the unweighted model. Associations are labeled as not significant (NS), concordant direction and significant (C + S (replicated)), concordant direction and not significant (C + NS), discordant direction and significant (D + S), and discordant direction and not significant (D + NS).

(E) Replication across model schemes for variant-phecode associations across 23 phecodes. The number in parentheses indicates the number of queried associations per phecode.

weighted setting. Among the associations that replicated in the weighted model setting but failed to replicate in the unweighted setting were several well-established associations; for example, variants in *PPARG* implicated in type 2 diabetes[56] (intronic variant rs11709077, weighted p = 2.57e−5, unweighted p = 0.076; missense variant rs1801282, weighted p = 1.39e−4, unweighted p = 0.12) and in the *CELSR2-PRSC1-SORT1* gene cluster, associated with coronary atherosclerosis[57] (rs7528419 lies in the 3′ UTR of *CELSR2*, weighted p = 3.47e−6, unweighted p = 0.10). The number of replicated associations in the weighted setting was significantly greater than in the unweighted setting: when considering the associations that were significant in only one of the two schemes (chi-squared test = 23.45, p = 3.49e−8; Figure 3A), or when considering all significant associations including those significant in both schemes (chi-squared test = 44.38, p = 3.29e−5, degrees of freedom [df] = 1). Moreover, when considering associations that replicated in both settings, the weighted setting yielded smaller p values on average (Figure 3B). Not all associations found to be significant in our sample had a consistent direction of effect with the *pgrm* catalog—notably, the weighted setting introduced more significant associations with opposing direction of effect (n = 114, 6% of total tested associations compared to the 0.2% of tested associations in the unweighted setting (n = 3), chi-squared test = 106.74, p < 2.2e−16, df = 1) (Figure 3C). Of these 114 associations, 68 (3.6% of the

1,879 tested associations) also had discordant direction of effect in the unweighted setting, with 2 associations meeting the significance threshold in the unweighted setting. On the other hand, 46 associations had concordant direction of effect in the unweighted setting, but only 6 of these (0.32% of associations tested) were significant, albeit nominally ($p > 0.008$; Figure 3D).

Conversely, none of the three unweighted associations with discordant effect with pgrm were replicated in the weighted setting.

While rarely observed, this discordance in direction between effect sizes from the unweighted and weighted model settings can arise from heterogeneity in effect sizes across strata of the weight distribution. We observed this phenomenon in the 6 significant and discordant associations from the weighted model setting that were replicated in the unweighted model. In these cases, a discordant direction of effect among individuals in the tail of the weight distribution drives an overall discordant weighted effect, while the corresponding unweighted association remains positive (Figure S9).

One factor that may contribute to such stratification of effect sizes is ancestry-specific effects. As expected, genetic ancestry is not uniformly distributed across quartiles of the weight distribution, with a larger proportion of individuals of non-EUR ancestry represented in higher weight quartiles (i.e., lower enrollment probability). Furthermore, ancestry-specific analyses reveal a higher frequency of discordant associations among individuals of non-EUR ancestry, which, when upweighted, can drive discordant effects in aggregate trans-ancestry analyses (Figure S10). As a sensitivity analysis, we performed a meta-analysis of ancestry-specific associations across five genetic ancestries as aligned to the 1000 Genomes reference (AFR: $n = 2{,}502$, AMR: $n = 7{,}247$, EAS: $n = 4{,}549$, EUR: $n = 30{,}472$, SAS: $n = 736$, and unassigned/ambiguous: $n = 3{,}158$). This analysis revealed trends consistent with the mega-analysis results, with greater replication observed in the weighted model setting (20.3% of associations replicated in both model settings, 11.1% in the unweighted-only setting, and an additional 16.1% in the weighted-only setting). Within each ancestry group, the weighted model setting replicated more associations than the unweighted (Table S6). Also, a similar subset of associations (5.5% of all tested associations) emerged as significant with opposing directions of effect across model settings (Table S6). These discordant associations largely overlapped with those identified in the mega-analysis. Specifically, the 6 significant discordant associations observed in the weighted model remained discordant in this analysis, albeit less significantly so.

The increased replication and trends in strength and direction of effect are robust to varying p value thresholds for significance (Figure S11; Table S5) and remain unchanged when weights are generated using the full random forest model probabilities instead of those generated by the small 5-feature model (Figure S12). Replicated associations were observed across 23 phecodes for both weighting schemes (Figure 3D), with a single association with hematuria that replicated only in the unweighted model setting and two associations with pneumonia and insomnia that replicated only in the weighted setting.

Weighting scheme modifies observed phenotypic relationships in phenome-wide associations with polygenic risk scores

We performed weighted and unweighted phenome-wide scans because we hypothesize that associations significant in both unweighted and weighted models may indicate more reliable associations in the ATLAS biobank. We tested a collection of 1,117 phecodes with five PGSs: MDD, BMI, RA, T1D, and general happiness with one's own health.

As expected, on-target phecodes were significantly associated with their corresponding PGS: "major depressive disorder" (296.22) with the MDD PGS (beta = 0.04, $p = 7.8e{-}79$), "overweight, obesity, and other hyperalimentation" (278) with the BMI PGS (beta = 0.07, $p = 3.2e{-}234$), "rheumatoid arthritis" (714.1) with the RA PGS (beta = 0.008, $p = 3.98e{-}19$), and "type 1 diabetes mellitus" (250.1) with the T1D PGS (beta = 0.01, $p = 4.16e{-}60$). The effect of the weighting scheme was minimal on these associations (Table S7). Across all PGSs, we observed that the unweighted setting identified more significant associations than the weighted setting, but the number of associations significant in unweighted, weighted, or both settings varied by trait (Figures 4 and S13; Tables S7 and S8). For MDD, BMI, and RA PGSs, the majority of associations were significant in both the unweighted and weighted settings, suggesting a minimal effect of inclusion biases. This was true especially in the mood disorders chapter for MDD and in the musculoskeletal chapter for RA. For happiness with one's own health, on the other hand, the majority of associations found to be significant were in the unweighted setting and were not corroborated under the weighting scheme. For T1D, few associations were found in both model schemes, but the majority of significant associations were found only in weighted models. Still, the "on-target" category of endocrine and metabolic phecodes was significant in both weighting schemes. We present a breakdown of phecode categories that includes phecodes found to be significant only in weighted PheWAS in Figure S14. These findings were similar when using predicted probabilities from the full random forest model instead of the 5-feature model (Figure S15).

To compare our enrollment-aware weighting scheme with direct covariate adjustment, we adjusted the unweighted model for the 5 features that were used to generate enrollment probability estimates. For all traits, this direct adjustment had more of an effect on associations found to be significant only in the unweighted setting, compared to those found significant in both

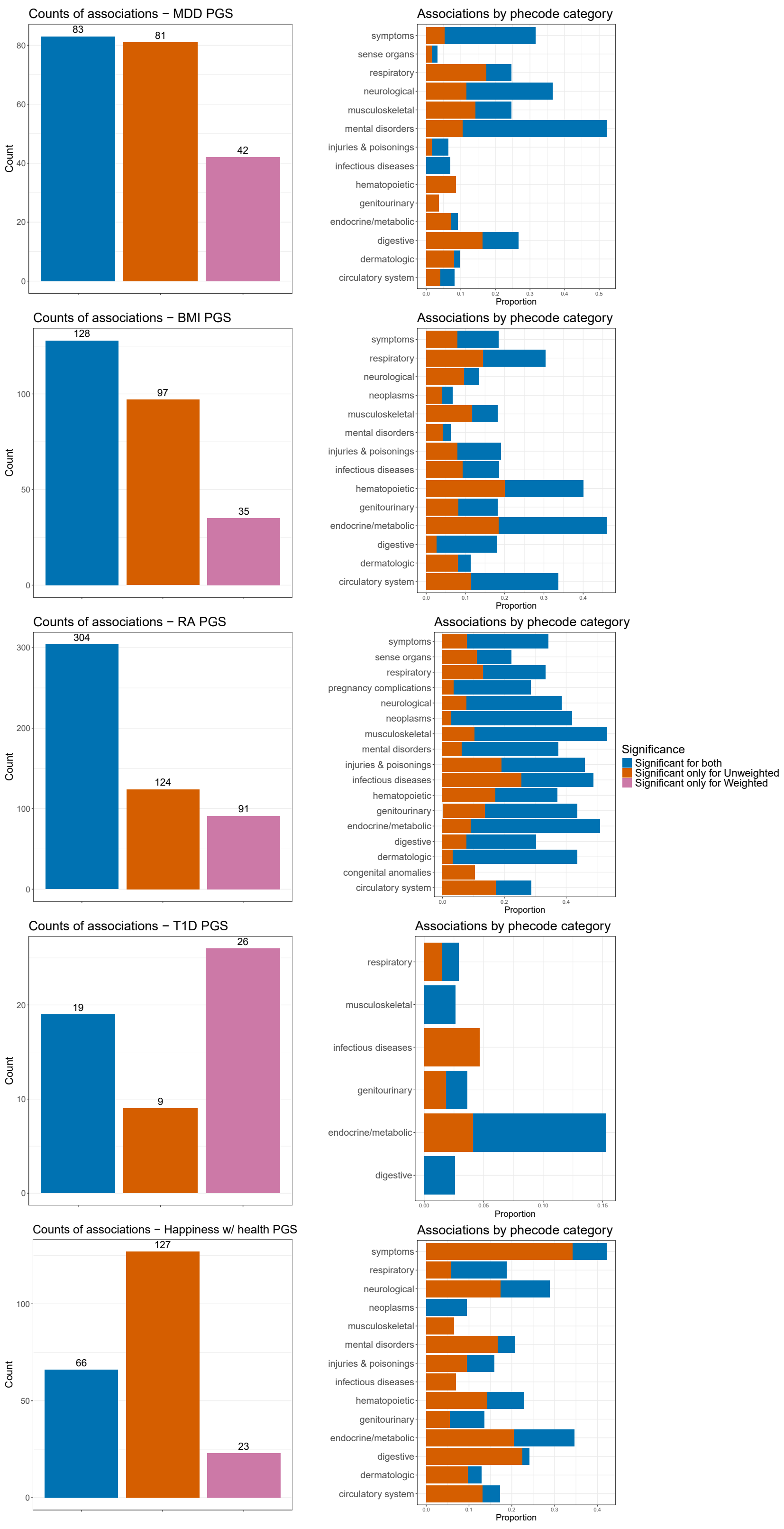

Figure 4. Comparison of unweighted and weighted PGS-PheWAS associations for 5 PGS

Overall (left) and phecode-stratified (right) counts of shared and unique associations under the unweighted and weighted model schemes for MDD, BMI, RA, T1D, and happiness with one's own health PGSs. Significance was assessed using a Bonferroni-corrected p value threshold of $p < 4.44e{-}5$ for 1,117 phecodes tested.

unweighted and weighted models (Table S9). For example, across the 97 phecodes found significantly associated with the BMI PGS in the unweighted setting alone, 55 (56.7%) became non-significant after direct adjustment. On the other hand, across the 128 associations that were significant under both the unweighted and weighted settings, only 26 (20.3%) became non-significant, while the remaining associations were robust to direct adjustment. These findings are consistent with the possibility that some of the associations observed as significant in the unweighted analysis could reflect bias in the biobank. We observed a heterogeneous pattern of effect sizes for each of the 5 features across PGS-PheWAS associations, indicating that the bias induced by these features may vary by phecode and PGS and go beyond their association with enrollment (Figure S16; Table S10).

Discussion

Quantifying the origin of inclusion biases and their impact on genetic analyses and developing analytic mitigation strategies are crucial for making accurate inferences from biobank studies. We assessed inclusion bias in the UCLA ATLAS biobank, which enrolls participants from the UCLA Health System. We found that healthcare utilization, indices of social determinants of health, and ICD-10 codes for symptoms and general healthcare encounters distinguished ATLAS participants from the background UCLA Health System. Our findings suggest that correcting for these features via inverse probability adjustment, despite decreasing ESS, increases the power for genetic analyses and may be used to detect false positive associations.

Factors found to be associated with enrollment, including self-identified sex, healthcare utilization, and socioeconomic status, were consistent with previous findings in population-based cohorts such as the UK Biobank[21] and EHR-linked biobanks such as the Mass General Brigham Biobank[24] and were stable across various sensitivity analyses. Notably, missing

demographic information, such as smoking status and ethnoracial category, was also associated with enrollment. These features were recovered from individual self-report surveys, where missingness has previously been shown to be associated with participation bias.[58]

We observed increased replication of known phenotype-genotype associations when using inverse probability reweighting, which suggests increased power when adjusting for inclusion biases. Notably, weighting recovered functionally relevant associations, such as those involving the *PPARG*[56] and *CELSR2*[57] genes and genomic regions linked to diabetes and coronary atherosclerosis.

It is important to note that these analyses are performed using GWASs conducted in populations of primarily EUR genetic ancestry, which may limit portability in ATLAS, a diverse biobank with greater representation of diverse ancestry groups. Since we observed that individuals of non-EUR ancestry have higher weights than EUR individuals and thus have an increased contribution in the weighted analysis, it may be surprising that more associations were replicated under the bias-aware weighted model setting. This suggests that, beyond ancestry differences, additional sources of bias significantly affect association outcomes.

However, we also observed a handful of significant associations with discordant directions of effect with the *pgrm* catalog that replicated in the unweighted setting. These associations exhibit ancestry-specific effects that, when analyzed in aggregate, have increased contributions due to the weighting scheme. These discordant effects remain, albeit not always significantly so, when meta-analyzing effect sizes across associations performed within ancestry groups. Notably, similar to the mega-analysis in the full sample, analyses within ancestry groups, as well as cross-ancestry meta-analyses, also demonstrate an increased replication rate in the weighted setting.

Previous work has shown that adjusting for inclusion bias using inverse probability weighting[21] or model-based formulations that account for genetic and non-genetic components underlying enrollment[59] affects genetic correlations and heritability estimation. Associations with BMI and alcohol consumption,[22] as well as calibration of predictive intervals of PGSs,[60] have been shown to be highly dependent on factors such as socioeconomic context. Different from previous studies, we now demonstrate that this also affects variant-level associations. We also expand on the analysis of the effect of inclusion bias on associations with PGSs and show that PGS associations are impacted by inclusion bias across the phenome.

It is important to note that adjustment for bias in this work is done in an enrollment-aware manner, where we aim to understand the contribution of these contextual measures on one specific potential confounder: enrollment behavior. While inverse probability reweighting may have different effects on specific PGSs and phenotypes, the heterogeneous effect of direct adjustment for relevant covariates indicates that weights directly informed by recorded enrollment outcomes may provide a flexible and generalizable framework for adjustments in downstream modeling tasks. In fact, accounting for bias on a case-by-case basis might be useful to contextualize the effects of covariates in each association, but it entails significant analysis and may capture additional sources of bias independent of enrollment that may not apply uniformly in phenome-wide study contexts. Unexpected associations identified through weighted analyses, such as those that change direction compared to unweighted analyses, can be further interrogated by evaluating stratification across the weight distribution and by testing specific bias-inducing features that may interact with such associations. We also note that the framework used here will not be suitable for rare variant studies, as reweighting variants with low frequency may further limit power by attenuating sample size and may dramatically skew associations.[61]

Directions for future work

Our work highlights several directions for future work. First, we focused on a single and simple reweighting scheme, which, due to the use of linear models to ensure convergence, prevents the direct comparison of effect sizes with logistic regression models.[43,44] Other strategies to account for sources of bias should be explored, as they could lead to different results and also enable the analysis of rare variants. In particular, in the proposed modeling scheme, the tails of the weight distribution can have a large impact on associations, and future work could explore ways to refine weighting schemes to better account for extreme weights. Second, while results showed stability of feature importance over time, as the ATLAS Initiative continues to grow toward its initial target of 150,000 participants,[26,27] this analysis will have to be reassessed in the future. Insights from these analyses may potentially inform future recruitment strategies in ATLAS and other biobanks. Furthermore, UCLA ATLAS is one in a growing collection of health-system-linked biobanks, and while our findings may be shared in other biobanks, additional studies are vital to assess generalizability. Third, comparing biobank participants with individuals served by the same health system has the advantage of rich data being available without the need for data harmonization. ATLAS is one of the most diverse biobanks in terms of genetic ancestry,[6,27] serving the metropolitan area of Los Angeles. However, in this work, we did not attempt to compare ATLAS to the local Los Angeles community or broader reference populations (e.g., city, county, or national population), as is done in previous work.[21] Choosing a shared background population as a reference could be a way of harmonizing across biobanks in aggregated study settings. Other strategies for cross-biobank harmonization, such as meta-analyzing enrollment in any biobank as an outcome, can also be used to build predictive models of enrollment that can be applied broadly. Finally, it is essential to note that inclusion bias encompasses both recruitment and

participation biases. Recruitment bias refers to the process of offering enrollment to individuals, while participation bias refers to individuals' acceptance of the enrollment offer. Observationally, these effects are difficult to separate without additional information; data recording those who were offered enrollment and declined were unavailable to us. Future work will involve the comparison of individuals who opted into the biobank to those who explicitly declined enrollment to disentangle biases induced by recruitment and participant opt-in decisions.

Taken together, this work emphasizes the importance of considering inclusion biases in volunteer cohort study settings, with a focus on contextualizing discoveries that impact clinical care settings. Accounting for factors related to enrollment can inform recruitment protocols while also allowing for explicit post hoc model adjustment in genetic investigations. With the growing utilization of genetic results in clinical contexts for screening and the return of actionable results, inferences drawn from volunteer populations ought to be contextualized and calibrated to represent the background non-research participant population.

Data and code availability

Descriptive and summary statistics are contained within the manuscript and supplemental information. All individual-level patient records and genetic data are not available due to patient confidentiality and security concerns.

Acknowledgments

The research reported here was supported by the National Library of Medicine (NLM) T15 LM013976 (to A.P.), National Institute of Mental Health (NIMH) R00 MH116115 (to L.M.O.L.), R01 NIMH MH137219 (to L.M.O.L.), NIMH U01 MH125042 (to L.M.O.L.), and the National Institutes of Health (NIH) U01 HG011715 (to B.P.). We gratefully acknowledge the support of the Institute for Precision Health, participating individuals from the UCLA ATLAS Precision Health Biobank, UCLA David Geffen School of Medicine, UCLA Clinical and Translational Science Institute grant number UL1TR001881, and UCLA Health. The UCLA ATLAS Community Health Initiative, in collaboration with the UCLA ATLAS Precision Health Biobank, is a program of IPH, which directs and supports the biobanking and genotyping of biospecimen samples from participating UCLA patients in collaboration with the David Geffen School of Medicine, UCLA CTSI, and UCLA Health. Additionally, we greatly acknowledge Daniel H. Geschwind, Clara Lajonchere, and Maryam Ariannejad for their insightful feedback.

Declaration of interests

The authors declare no competing interests.

Supplemental information

Supplemental information can be found online at https://doi.org/10.1016/j.ajhg.2026.02.011.

Received: April 2, 2025
Accepted: February 9, 2026
Published: March 10, 2026

References

1. Bowton, E., Field, J.R., Wang, S., Schildcrout, J.S., Van Driest, S.L., Delaney, J.T., Cowan, J., Weeke, P., Mosley, J.D., Wells, Q.S., et al. (2014). Biobanks and electronic medical records: enabling cost-effective research. Sci. Transl. Med. *6*, 234cm3. https://doi.org/10.1126/scitranslmed.3008604.
2. Wolford, B.N., Willer, C.J., and Surakka, I. (2018). Electronic health records: the next wave of complex disease genetics. Hum. Mol. Genet. *27*, R14–R21. https://doi.org/10.1093/hmg/ddy081.
3. Phung, L., Wood, E., Egleston, B., Hoffman-Andrews, L., Ofidis, D., Howe, S., Mim, R., Griffin, H., Fetzer, D., Owens, A., et al. (2024). Facilitating return of actionable genetic research results from a biobank repository: Participant uptake and utilization of digital interventions. HGG Adv. *5*, 100346. https://doi.org/10.1016/j.xhgg.2024.100346.
4. Lewis, C.M., and Vassos, E. (2020). Polygenic risk scores: from research tools to clinical instruments. Genome Med. *12*, 44. https://doi.org/10.1186/s13073-020-00742-5.
5. Clayton, E.W., Smith, M.E., Anderson, K.C., Chung, W.K., Connolly, J.J., Fullerton, S.M., McGowan, M.L., Peterson, J.F., Prows, C.A., Sabatello, M., and Holm, I.A. (2023). Studying the impact of translational genomic research: Lessons from eMERGE. Am. J. Hum. Genet. *110*, 1021–1033. https://doi.org/10.1016/j.ajhg.2023.05.011.
6. Abul-Husn, N.S., and Kenny, E.E. (2019). Personalized medicine and the power of electronic health records. Cell *177*, 58–69. https://doi.org/10.1016/j.cell.2019.02.039.
7. Khera, A.V., Chaffin, M., Aragam, K.G., Haas, M.E., Roselli, C., Choi, S.H., Natarajan, P., Lander, E.S., Lubitz, S.A., Ellinor, P.T., and Kathiresan, S. (2018). Genome-wide polygenic scores for common diseases identify individuals with risk equivalent to monogenic mutations. Nat. Genet. *50*, 1219–1224. https://doi.org/10.1038/s41588-018-0183-z.
8. Lambert, S.A., Abraham, G., and Inouye, M. (2019). Towards clinical utility of polygenic risk scores. Hum. Mol. Genet. *28*, R133–R142. https://doi.org/10.1093/hmg/ddz187.
9. Mars, N., Koskela, J.T., Ripatti, P., Kiiskinen, T.T.J., Havulinna, A.S., Lindbohm, J.V., Ahola-Olli, A., Kurki, M., Karjalainen, J., Palta, P., et al. (2020). Polygenic and clinical risk scores and their impact on age at onset and prediction of cardiometabolic diseases and common cancers. Nat. Med. *26*, 549–557. https://doi.org/10.1038/s41591-020-0800-0.
10. Aguirre, M., Tanigawa, Y., Venkataraman, G.R., Tibshirani, R., Hastie, T., and Rivas, M.A. (2021). Polygenic risk modeling with latent trait-related genetic components. Eur. J. Hum. Genet. *29*, 1071–1081. https://doi.org/10.1038/s41431-021-00813-0.
11. Chen, J., Mize, T., Wu, J.-S., Hong, E., Nimgaonkar, V., Kendler, K.S., Allen, D., Oh, E., Netski, A., and Chen, X. (2020). Polygenic risk scores for subtyping of schizophrenia. Schizophr. Res. Treatment *2020*, 1638403. https://doi.org/10.1155/2020/1638403.
12. Lu, Y., Kowalec, K., Song, J., Karlsson, R., Harder, A., Giusti-Rodríguez, P., Sullivan, P.F., and Yao, S. (2023). Subtyping schizophrenia using psychiatric polygenic scores. Preprint at medRxiv. https://doi.org/10.1101/2023.10.12.23296915.

13. Yin, L., Lin, Y., Lui, S.S.-Y., and So, H.-C. (2023). Incorporating clinical, genomic profiles and polygenic risk scores for the subtyping of depressive disorders. Preprint at medRxiv. https://doi.org/10.1101/2023.03.01.23286610.
14. Mega, J.L., Stitziel, N.O., Smith, J.G., Chasman, D.I., Caulfield, M., Devlin, J.J., Nordio, F., Hyde, C., Cannon, C.P., Sacks, F., et al. (2015). Genetic risk, coronary heart disease events, and the clinical benefit of statin therapy: an analysis of primary and secondary prevention trials. Lancet *385*, 2264–2271. https://doi.org/10.1016/S0140-6736(14)61730-X.
15. Mateo, J., Steuten, L., Aftimos, P., André, F., Davies, M., Garralda, E., Geissler, J., Husereau, D., Martinez-Lopez, I., Normanno, N., et al. (2022). Delivering precision oncology to patients with cancer. Nat. Med. *28*, 658–665. https://doi.org/10.1038/s41591-022-01717-2.
16. Pedersen, E.M., Wimberley, T., and Vilhjálmsson, B.J. (2024). A cautionary tale for Alzheimer's disease GWAS by proxy. Nat. Genet. *56*, 2590–2591. https://doi.org/10.1038/s41588-024-02023-y.
17. Isgut, M., Giuste, F., Gloster, L., Swain, A., Choi, K., Hornback, A., Deshpande, S.R., and Wang, M.D. (2024). Identifying and characterizing disease subpopulations that most benefit from polygenic risk scores. Sci. Rep. *14*, 22124. https://doi.org/10.1038/s41598-024-63705-5.
18. Fry, A., Littlejohns, T.J., Sudlow, C., Doherty, N., Adamska, L., Sprosen, T., Collins, R., and Allen, N.E. (2017). Comparison of sociodemographic and health-related characteristics of UK Biobank participants with those of the general population. Am. J. Epidemiol. *186*, 1026–1034. https://doi.org/10.1093/aje/kwx246.
19. van Alten, S., Domingue, B.W., Faul, J., Galama, T., and Marees, A.T. (2024). Reweighting UK Biobank corrects for pervasive selection bias due to volunteering. Int. J. Epidemiol. *53*, dyae054. https://doi.org/10.1093/ije/dyae054.
20. Stamatakis, E., Owen, K.B., Shepherd, L., Drayton, B., Hamer, M., and Bauman, A.E. (2021). Is cohort representativeness passé? Poststratified associations of lifestyle risk factors with mortality in the UK biobank. Epidemiology *32*, 179–188. https://doi.org/10.1097/ede.0000000000001316.
21. Schoeler, T., Speed, D., Porcu, E., Pirastu, N., Pingault, J.-B., and Kutalik, Z. (2023). Participation bias in the UK Biobank distorts genetic associations and downstream analyses. Nat. Hum. Behav. *7*, 1216–1227. https://doi.org/10.1038/s41562-023-01579-9.
22. Shapland, C.Y., Gkatzionis, A., Hemani, G., and Tilling, K. (2025). Use of genetic correlations to examine selection bias. Genet. Epidemiol. *49*, e22584. https://doi.org/10.1002/gepi.22584.
23. Rodosthenous, R.S., Niemi, M.E.K., Kallio, L., Perala, M., Terho, P., Knopp, T., Punkka, E., Makkonen, E.M., Nurmi, P., Makela, J., et al. (2022). Recontacting biobank participants to collect lifestyle, behavioural and cognitive information via online questionnaires: lessons from a pilot study within FinnGen. BMJ Open *12*, e064695. https://doi.org/10.1136/bmjopen-2022-064695.
24. Lee, Y.H., Thaweethai, T., Sheu, Y.-H., Feng, Y.-C.A., Karlson, E.W., Ge, T., Kraft, P., and Smoller, J.W. (2023). Impact of selection bias on polygenic risk score estimates in healthcare settings. Psychol. Med. *53*, 7435–7445. https://doi.org/10.1017/S0033291723001186.
25. Johnson, R., Ding, Y., Venkateswaran, V., Bhattacharya, A., Boulier, K., Chiu, A., Knyazev, S., Schwarz, T., Freund, M., Zhan, L., et al. (2022). Leveraging genomic diversity for discovery in an electronic health record linked biobank: the UCLA ATLAS Community Health Initiative. Genome Med. *14*, 104. https://doi.org/10.1186/s13073-022-01106-x.
26. Johnson, R., Ding, Y., Bhattacharya, A., Knyazev, S., Chiu, A., Lajonchere, C., Geschwind, D.H., and Pasaniuc, B. (2023). The UCLA ATLAS Community Health Initiative: Promoting precision health research in a diverse biobank. Cell Genom. *3*, 100243. https://doi.org/10.1016/j.xgen.2022.100243.
27. Haas, R., Margolis, M.P., Wei, A., Yamaguchi, T.N., Feng, J., Tran, T., Tozzo, V., Queen, K.J., Mootor, M.F.E., Patil, V., et al. (2025). Diverse genomes, shared health: Insights from a health system biobank. Preprint at medRxiv. https://doi.org/10.1101/2025.06.11.25329386.
28. Lajonchere, C., Naeim, A., Dry, S., Wenger, N., Elashoff, D., Vangala, S., Petruse, A., Ariannejad, M., Magyar, C., Johansen, L., et al. (2021). An integrated, scalable, electronic video consent process to power precision health research: Large, population-based, cohort implementation and scalability study. J. Med. Internet Res. *23*, e31121. https://doi.org/10.2196/31121.
29. United States Department of Health & Human Services. CDC/ATSDR's Social Vulnerability Index. https://www.atsdr.cdc.gov/place-health/php/svi/index.html.
30. Ong, P.M., Pech, C., Gutierrez, N.R., and Mays, V.M. (2021). COVID-19 medical vulnerability indicators: A predictive, local data model for equity in public health decision making. Int. J. Environ. Res. Public Health *18*, 4829. https://doi.org/10.3390/ijerph18094829.
31. Pedregosa, F., Varoquaux, G., Gramfort, A., Michel, V., Thirion, B., Grisel, O., Blondel, M., Prettenhofer, P., Weiss, R., Dubourg, V., et al. (2011). Scikit-learn: Machine learning in Python. J. Mach. Learn. Res. *12*, 2825–2830. http://jmlr.org/papers/v12/pedregosa11a.html.
32. Lundberg, S., and Lee, S.-I. (2017). A unified approach to interpreting model predictions. Preprint at arXiv. https://doi.org/10.48550/arXiv.1705.07874v2.
33. Lundberg, S.M., Erion, G., Chen, H., DeGrave, A., Prutkin, J.M., Nair, B., Katz, R., Himmelfarb, J., Bansal, N., and Lee, S.-I. (2020). From local explanations to global understanding with explainable AI for trees. Nat. Mach. Intell. *2*, 56–67. https://doi.org/10.1038/s42256-019-0138-9.
34. Zhang, L., Bujkiewicz, S., and Jackson, D. (2024). Three new methodologies for calculating the effective sample size when performing population adjustment. BMC Med. Res. Methodol. *24*, 287. https://doi.org/10.1186/s12874-024-02412-1.
35. Wiegand, H. (1968). Kish, L.: Survey sampling. John Wiley & sons, inc., New York, London 1965, IX + 643 S., 31 Abb., 56 tab., preis 83 s. Biom. Z. *10*, 88–89. https://doi.org/10.1002/bimj.19680100122.
36. Abraham, G., Qiu, Y., and Inouye, M. (2017). FlashPCA2: principal component analysis of Biobank-scale genotype datasets. Bioinformatics *33*, 2776–2778. https://doi.org/10.1093/bioinformatics/btx299.
37. 1000 Genomes Project Consortium, Auton, A., Brooks, L.D., Durbin, R.M., Garrison, E.P., Kang, H.M., Korbel, J.O., Marchini, J.L., McCarthy, S., McVean, G.A., et al. (2015). A global

reference for human genetic variation. Nature *526*, 68–74. https://doi.org/10.1038/nature15393.
38. Bastarache, L. (2021). Using phecodes for research with the electronic health record: From PheWAS to PheRS. Annu. Rev. Biomed. Data Sci. *4*, 1–19. https://doi.org/10.1146/annurev-biodatasci-122320-112352.
39. Wu, P., Gifford, A., Meng, X., Li, X., Campbell, H., Varley, T., Zhao, J., Carroll, R., Bastarache, L., Denny, J.C., et al. (2019). Mapping ICD-10 and ICD-10-CM codes to phecodes: Workflow development and initial evaluation. JMIR Med. Inform. *7*, e14325. https://doi.org/10.2196/14325.
40. Denny, J.C., Ritchie, M.D., Basford, M.A., Pulley, J.M., Bastarache, L., Brown-Gentry, K., Wang, D., Masys, D.R., Roden, D.M., and Crawford, D.C. (2010). PheWAS: demonstrating the feasibility of a phenome-wide scan to discover gene-disease associations. Bioinformatics *26*, 1205–1210. https://doi.org/10.1093/bioinformatics/btq126.
41. Bastarache, L., Delozier, S., Pandit, A., He, J., Lewis, A., Annis, A.C., LeFaive, J., Denny, J.C., Carroll, R.J., Altman, R.B., et al. (2023). The phenotype-genotype reference map: Improving biobank data science through replication. Am. J. Hum. Genet. *110*, 1522–1533. https://doi.org/10.1016/j.ajhg.2023.07.012.
42. Purcell, S., Neale, B., Todd-Brown, K., Thomas, L., Ferreira, M.A.R., Bender, D., Maller, J., Sklar, P., de Bakker, P.I.W., Daly, M.J., and Sham, P.C. (2007). PLINK: a tool set for whole-genome association and population-based linkage analyses. Am. J. Hum. Genet. *81*, 559–575. https://doi.org/10.1086/519795.
43. von Hippel, P. (2015). Linear vs. Logistic Probability Models: Which is Better, and When? Statistical Horizons. https://statisticalhorizons.com/linear-vs-logistic/.
44. von Hippel, P. (2017). When Can You Fit a Linear Probability Model? More Often Than You Think. Statistical Horizons. https://statisticalhorizons.com/when-can-you-fit/.
45. Gold, S.M., Köhler-Forsberg, O., Moss-Morris, R., Mehnert, A., Miranda, J.J., Bullinger, M., Steptoe, A., Whooley, M.A., and Otte, C. (2020). Comorbid depression in medical diseases. Nat. Rev. Dis. Primers *6*, 69. https://doi.org/10.1038/s41572-020-0200-2.
46. Viechtbauer, W. (2010). Conducting Meta-Analyses inRwith themetaforPackage. J. Stat. Softw. *36*, 1–48. https://doi.org/10.18637/jss.v036.i03.
47. Wang, J., Wu, X., Lai, W., Long, E., Zhang, X., Li, W., Zhu, Y., Chen, C., Zhong, X., Liu, Z., et al. (2017). Prevalence of depression and depressive symptoms among outpatients: a systematic review and meta-analysis. BMJ Open *7*, e017173. https://doi.org/10.1136/bmjopen-2017-017173.
48. Walker, J., Burke, K., Wanat, M., Fisher, R., Fielding, J., Mulick, A., Puntis, S., Sharpe, J., Esposti, M.D., Harriss, E., et al. (2018). The prevalence of depression in general hospital inpatients: a systematic review and meta-analysis of interview-based studies. Psychol. Med. *48*, 2285–2298. https://doi.org/10.1017/S0033291718000624.
49. Liu, N., Birstler, J., Venkatesh, M., Hanrahan, L., Chen, G., and Funk, L. (2021). Obesity and BMI cut points for associated comorbidities: Electronic health record study. J. Med. Internet Res. *23*, e24017. https://doi.org/10.2196/24017.
50. Mond, J.M., and Baune, B.T. (2009). Overweight, medical comorbidity and health-related quality of life in a community sample of women and men. Obesity *17*, 1627–1634. https://doi.org/10.1038/oby.2009.27.
51. Lambert, S.A., Gil, L., Jupp, S., Ritchie, S.C., Xu, Y., Buniello, A., McMahon, A., Abraham, G., Chapman, M., Parkinson, H., et al. (2021). The Polygenic Score Catalog as an open database for reproducibility and systematic evaluation. Nat. Genet. *53*, 420–425. https://doi.org/10.1038/s41588-021-00783-5.
52. Weissbrod, O., Kanai, M., Shi, H., Gazal, S., Peyrot, W.J., Khera, A.V., Okada, Y., Biobank Japan Project, Martin, A.R., Finucane, H.K., and Price, A.L. (2022). Leveraging fine-mapping and multipopulation training data to improve cross-population polygenic risk scores. Nat. Genet. *54*, 450–458. https://doi.org/10.1038/s41588-022-01036-9.
53. Als, T.D., Kurki, M.I., Grove, J., Voloudakis, G., Therrien, K., Tasanko, E., Nielsen, T.T., Naamanka, J., Veerapen, K., Levey, D.F., et al. (2023). Depression pathophysiology, risk prediction of recurrence and comorbid psychiatric disorders using genome-wide analyses. Nat. Med. *29*, 1832–1844. https://doi.org/10.1038/s41591-023-02352-1.
54. Lloyd-Jones, L.R., Zeng, J., Sidorenko, J., Yengo, L., Moser, G., Kemper, K.E., Wang, H., Zheng, Z., Magi, R., Esko, T., et al. (2019). Improved polygenic prediction by Bayesian multiple regression on summary statistics. Nat. Commun. *10*, 5086. https://doi.org/10.1038/s41467-019-12653-0.
55. Truong, H.P., Luke, A.A., Hammond, G., Wadhera, R.K., Reidhead, M., and Joynt Maddox, K.E. (2020). Utilization of social determinants of health ICD-10 Z-codes among hospitalized patients in the United States, 2016-2017. Med. Care *58*, 1037–1043. https://doi.org/10.1097/MLR.0000000000001418.
56. Sarhangi, N., Sharifi, F., Hashemian, L., Hassani Doabsari, M., Heshmatzad, K., Rahbaran, M., Jamaldini, S.H., Aghaei Meybodi, H.R., and Hasanzad, M. (2020). PPARG (Pro12Ala) genetic variant and risk of T2DM: a systematic review and meta-analysis. Sci. Rep. *10*, 12764. https://doi.org/10.1038/s41598-020-69363-7.
57. Hartmann, K., Seweryn, M., and Sadee, W. (2022). Interpreting coronary artery disease GWAS results: A functional genomics approach assessing biological significance. PLoS One *17*, e0244904. https://doi.org/10.1371/journal.pone.0244904.
58. Tyrrell, J., Zheng, J., Beaumont, R., Hinton, K., Richardson, T.G., Wood, A.R., Davey Smith, G., Frayling, T.M., and Tilling, K. (2021). Genetic predictors of participation in optional components of UK Biobank. Nat. Commun. *12*, 886. https://doi.org/10.1038/s41467-021-21073-y.
59. Song, S., Benonisdottir, S., Liu, J.S., and Kong, A. (2025). Participation bias in the estimation of heritability and genetic correlation. Proc. Natl. Acad. Sci. USA *122*, e2425530122. https://doi.org/10.1073/pnas.2425530122.
60. Hou, K., Xu, Z., Ding, Y., Mandla, R., Shi, Z., Boulier, K., Harpak, A., and Pasaniuc, B. (2024). Calibrated prediction intervals for polygenic scores across diverse contexts. Nat. Genet. *56*, 1386–1396. https://doi.org/10.1038/s41588-024-01792-w.
61. Chen, W., Coombes, B.J., and Larson, N.B. (2022). Recent advances and challenges of rare variant association analysis in the biobank sequencing era. Front. Genet. *13*, 1014947. https://doi.org/10.3389/fgene.2022.1014947.

The American Journal of Human Genetics, Volume *113*

Supplemental information

Inclusion bias affects common variant discovery and replication in a health-system linked biobank

Aditya Pimplaskar, Junqiong Qiu, Sandra Lapinska, Veronica Tozzo, Jeffrey N. Chiang, Bogdan Pasaniuc, and Loes M. Olde Loohuis

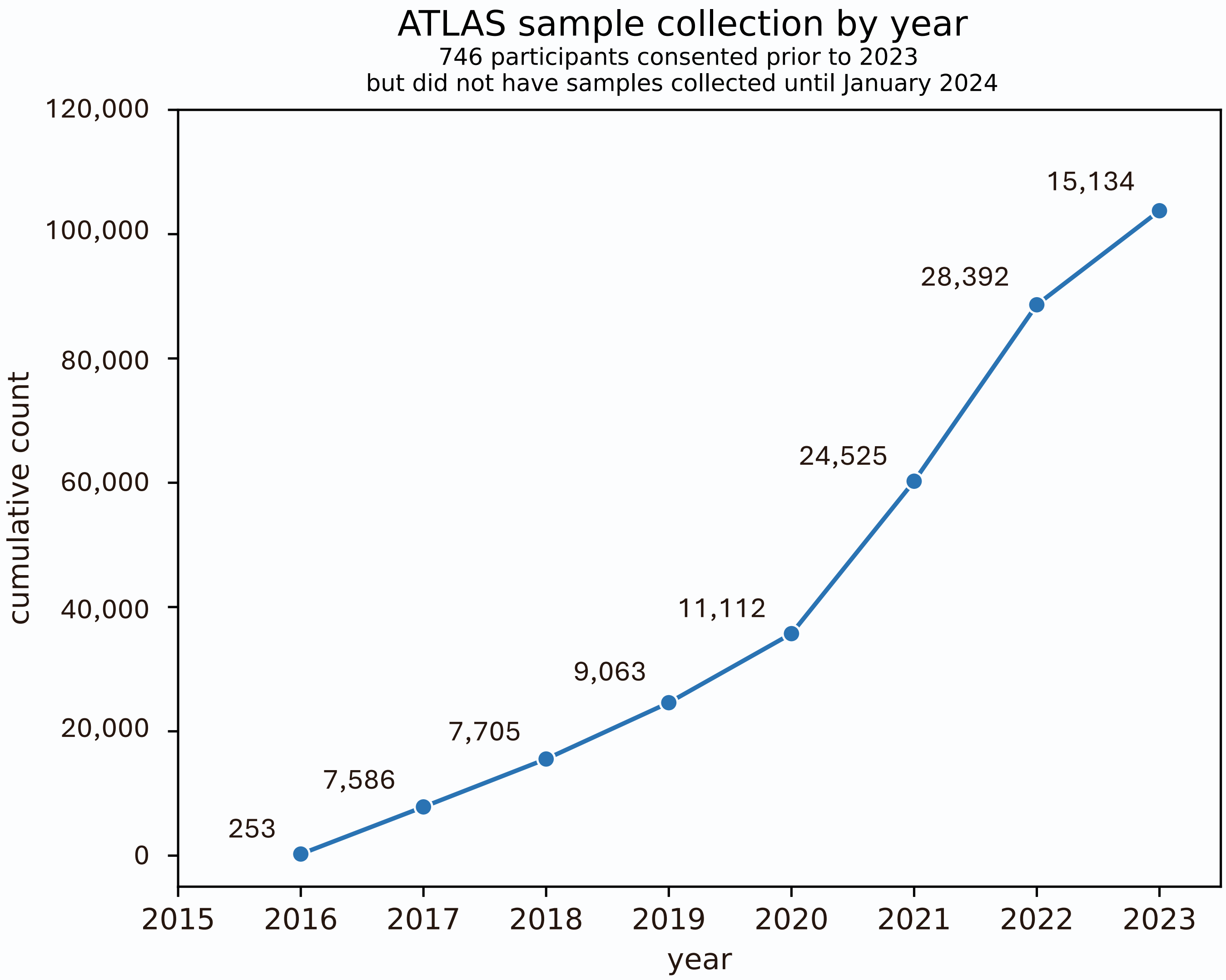

Figure S1. ATLAS sample collection counts by year: Cumulative and yearly counts of ATLAS sample collection by year, spanning enrollment for 2016-2023. A subset of individuals (n=746) individuals who consented into the biobank in 2023 but did not have samples collected until January of 2024 are not included in counts.

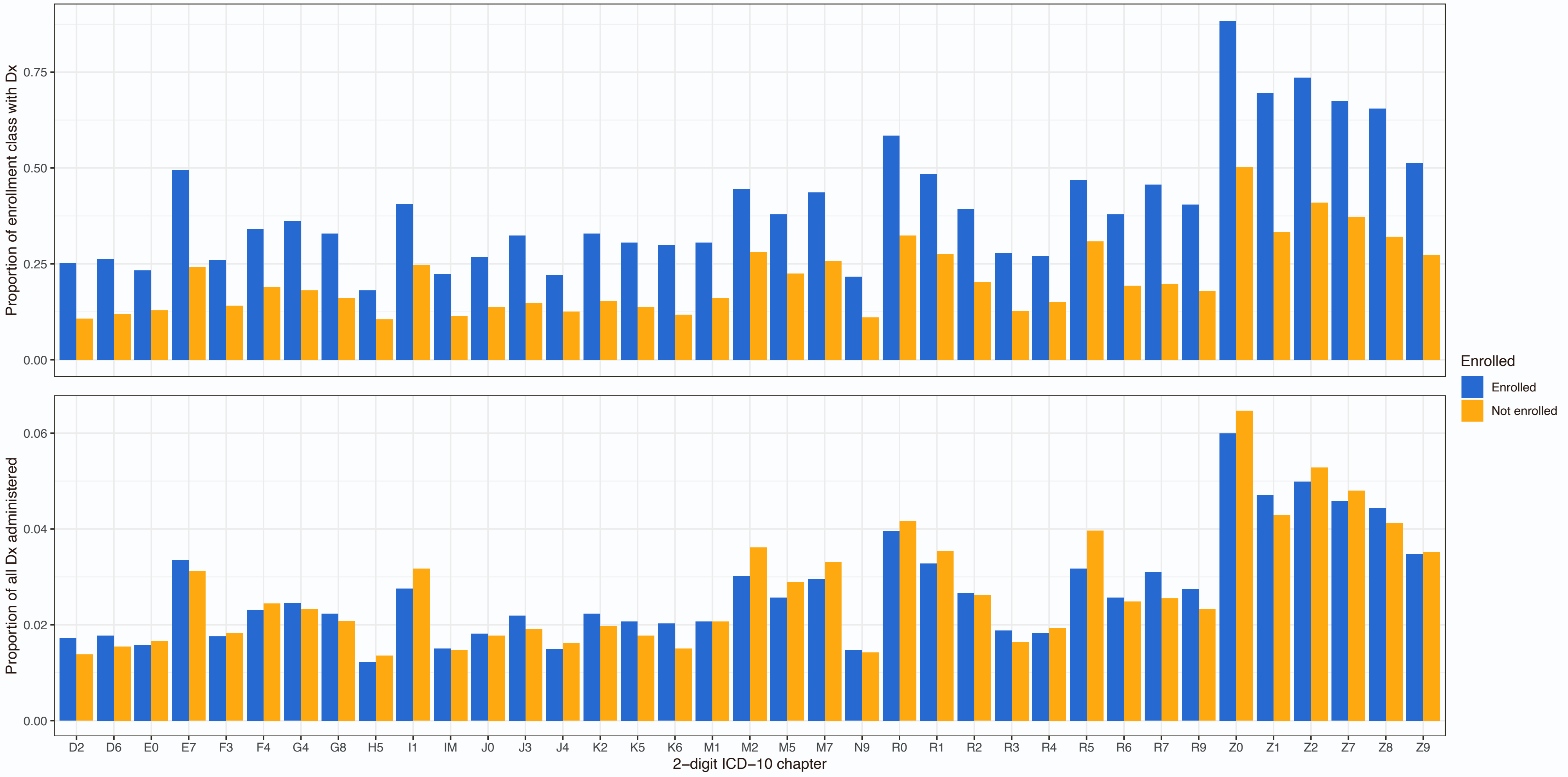

Figure S2. Comparison of diagnostic burden stratified by enrollment : Proportion of individuals stratified by enrollment with a given 2-digit ICD-10 diagnosis (top). Proportion of 2-digit ICD-10 diagnosis relative to all diagnoses administered within enrolled/unenrolled individuals (bottom).

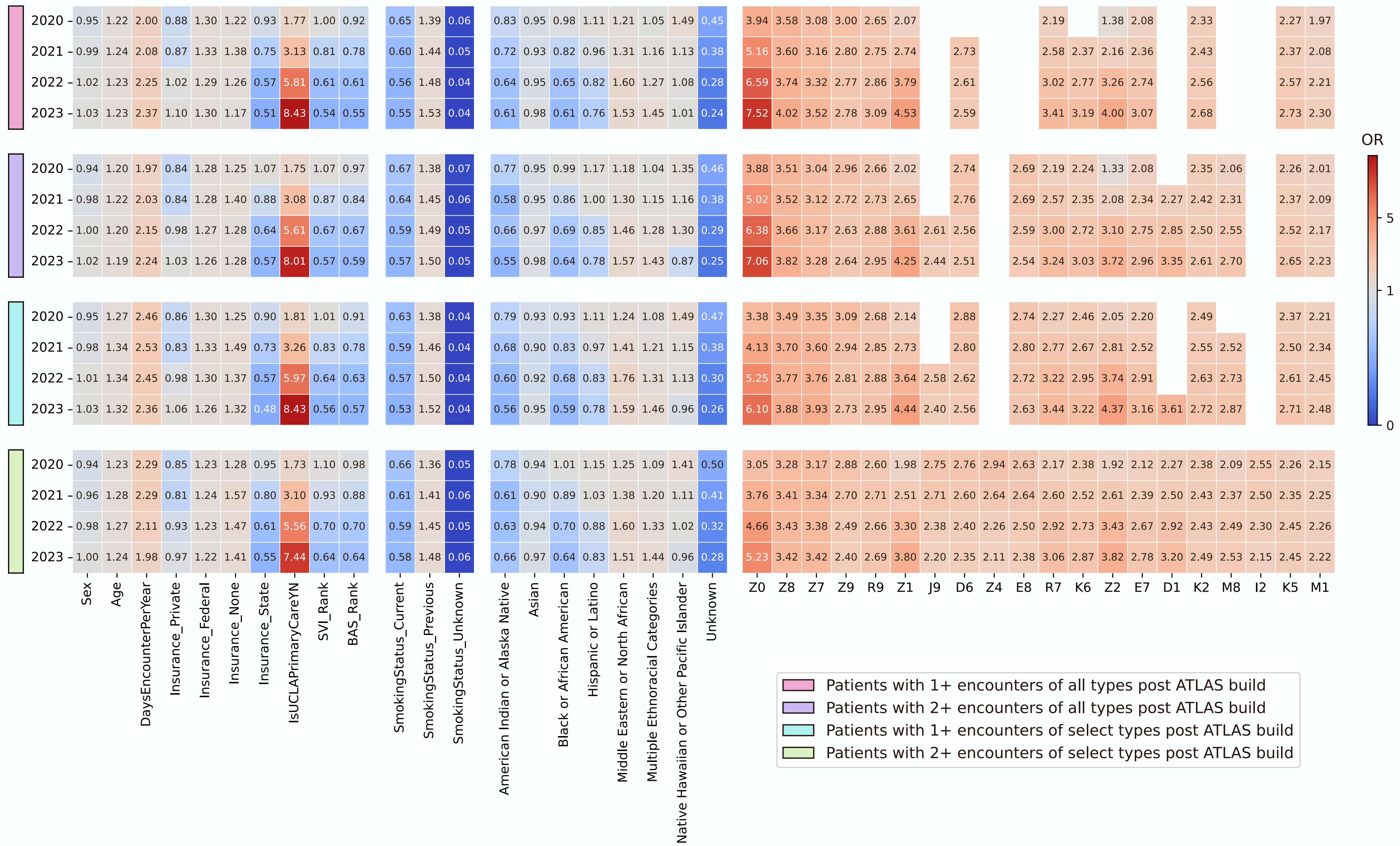

Figure S3. Comparison of feature level effects on enrollment across healthcare utilization inclusion criteria and aggregated across ATLAS recruitment timeframes: Feature level odds ratios compared across data horizons. For a given year, all individuals enrolled by that year were aggregated and compared to a reference consisting of unenrolled individuals up to the data horizon .

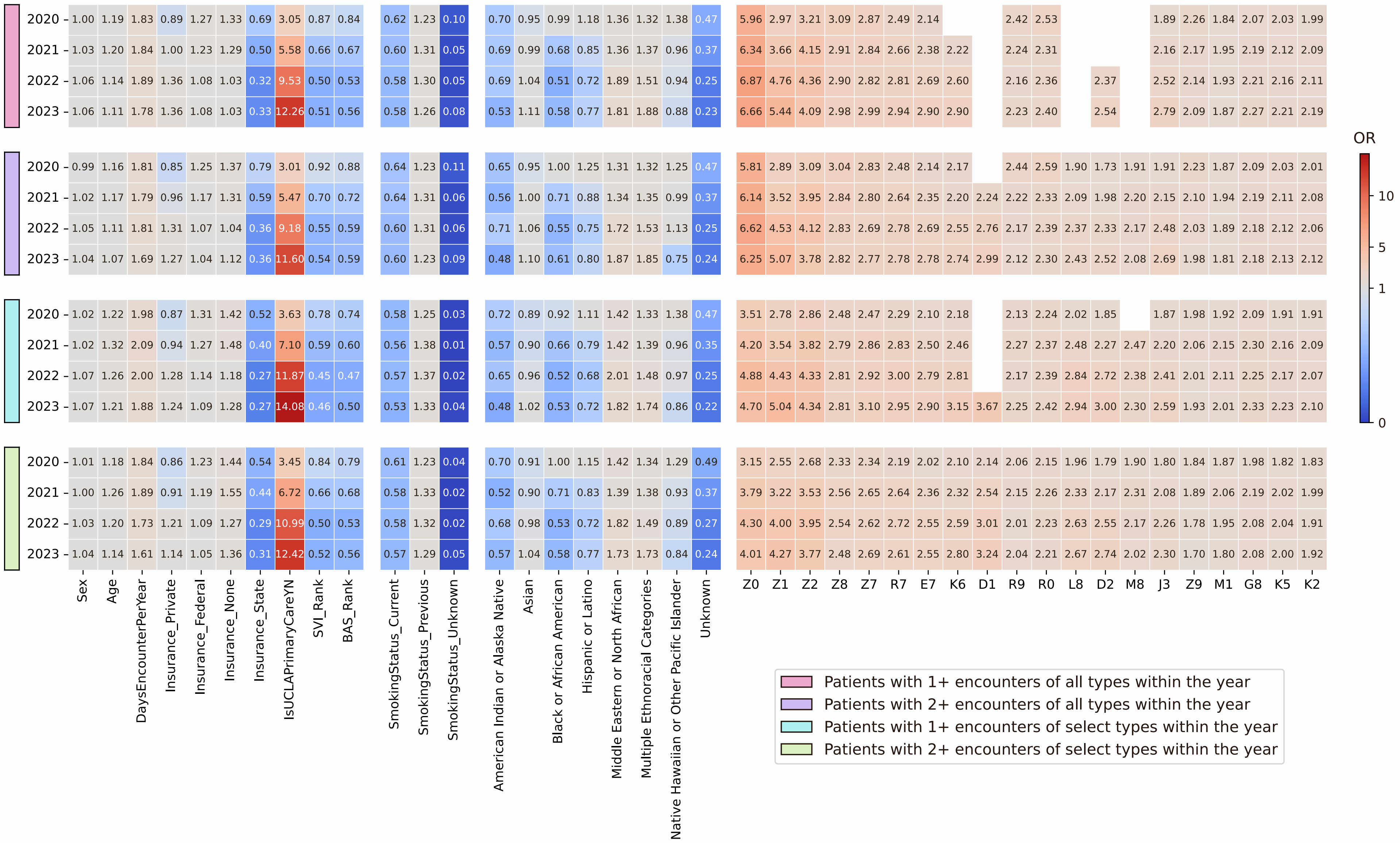

Figure S4. Comparison of per-year feature level effects on enrollment across healthcare utilization inclusion criteria: Feature level odds ratios compared across enrollment years. For a given year, all individuals enrolled by that year were aggregated and compared to a reference consisting of unenrolled individuals up to the data horizon. Individuals who had enrolled in years prior were omitted from the analysis.

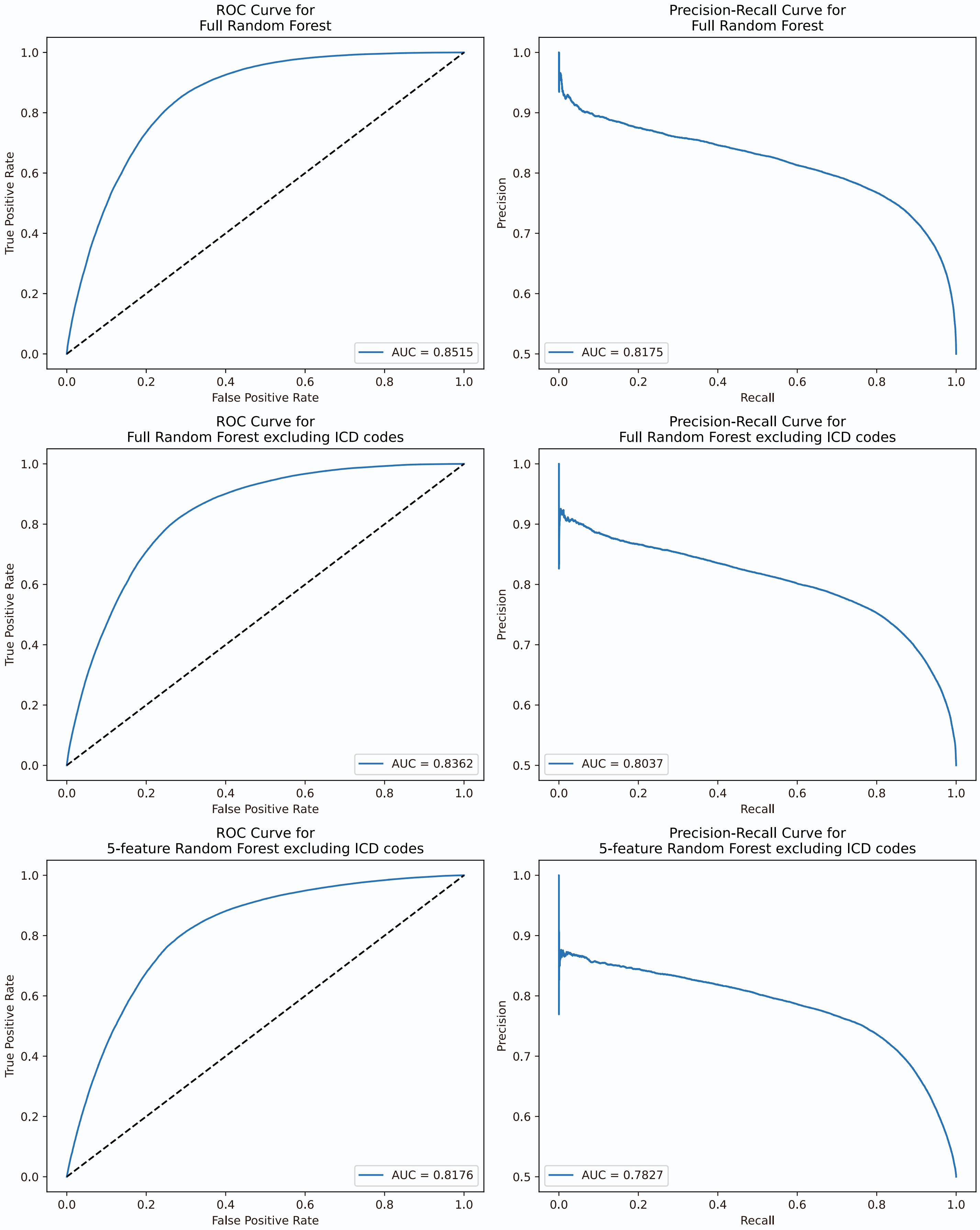

Figure S5. Random forest model performance across model settings: Assessment of random forest model performance using three different model formulations: full model, full model excluding ICD-10 codes, 5-feature recursive feature elimination

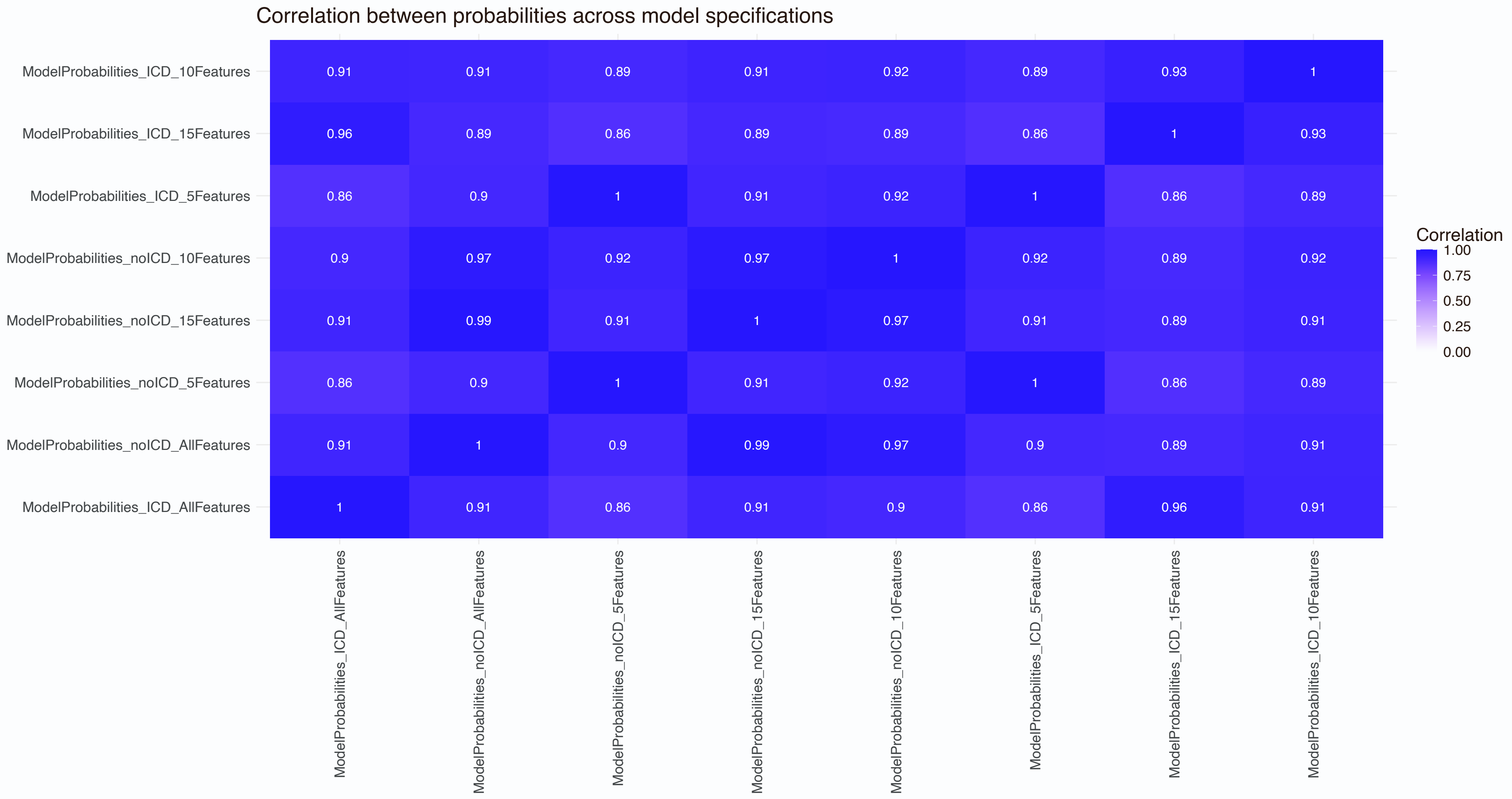

Figure S6. Correlation between enrollment probabilities across model specifications: Pearson correlation of enrollment probability varying recursive feature elimination cutoffs (5, 10, 15 features) and inclusion of ICD-10 codes in feature lists

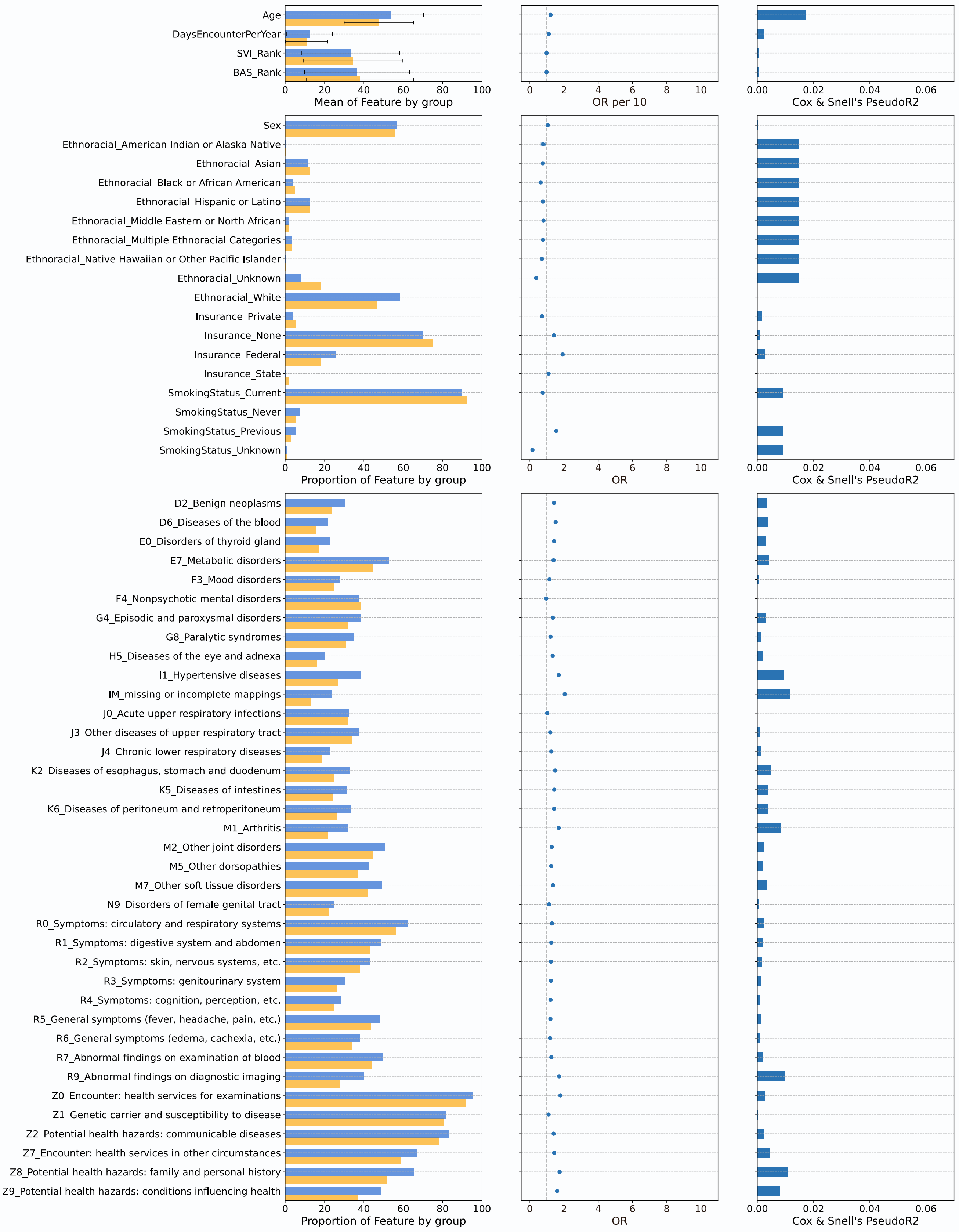

Figure S7. Feature distributions and univariate association statistics for enrollment in a subset of individuals receiving primary care at UCLA: (Left) Feature level distributions stratified by ATLAS enrollment (yellow = not enrolled in ATLAS, blue = enrolled in ATLAS) in a subset of patients receiving primary care at UCLA. For quantitative variables, feature means are depicted, and for categorical features, proportion of individuals in both groups in the feature group are depicted. For diagnostic chapters, any diagnosis in the chapter is sufficient. Reference levels for categorical features are as follows: Sex (male), Ethnoracial category (White), Smoking Status (Never). (Middle/right) Results from univariate associations with ATLAS enrollment in the UCLA Primary Care subset – Odds ratio (middle) and Cox & Snell's pseudo-R2 (right). Effect sizes for quantitative features are presented in the top panel as odds ratios per 10 units.

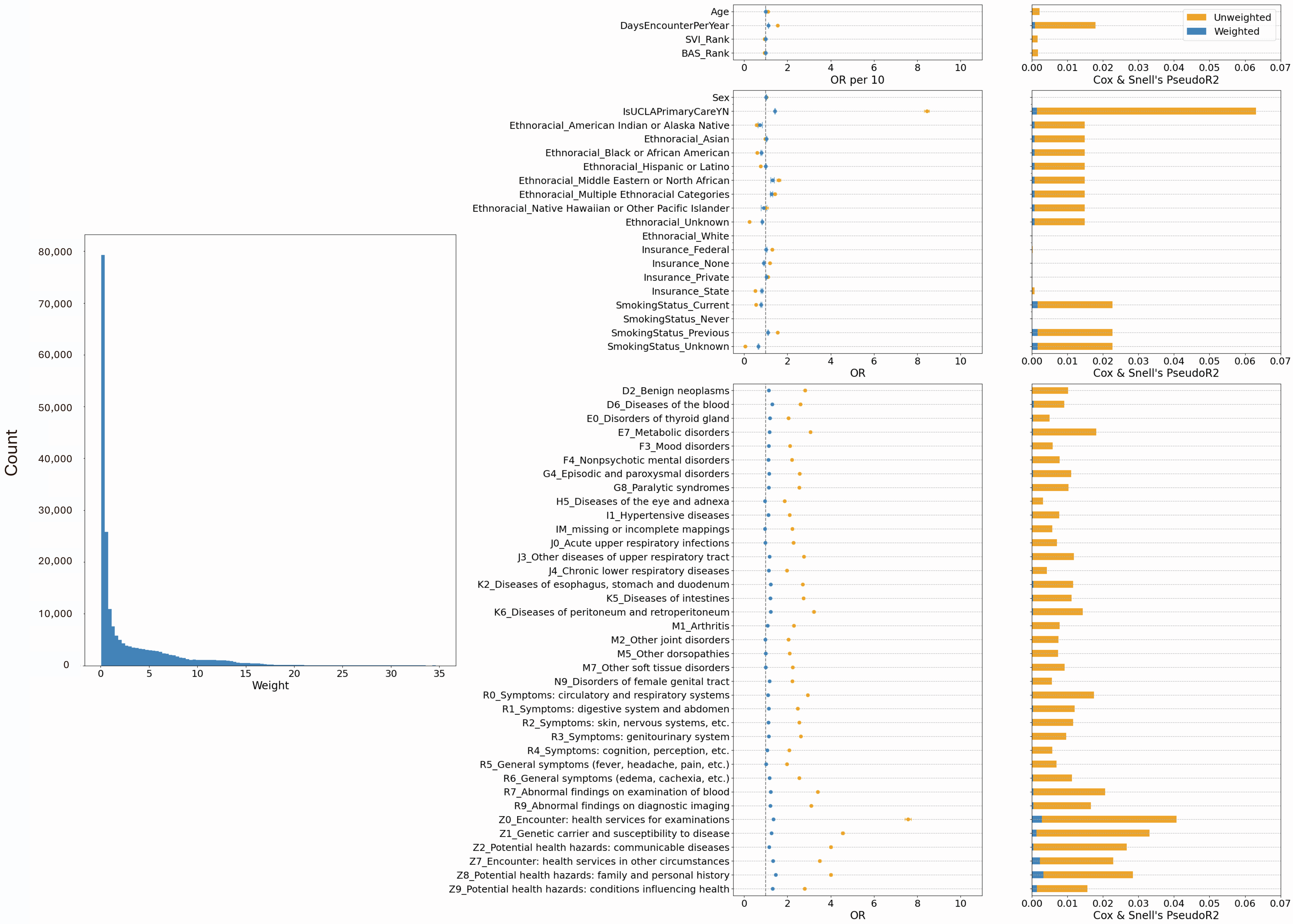

Figure S8. Weight distribution and effect of inverse-probability adjustment on univariate associations: Weights derived from transformation of enrollment probabilities using the 5-feature RFE model (left). Comparison of feature-level univariate effects and variance explained (right) between unweighted associations (blue) and weighted associations (orange).

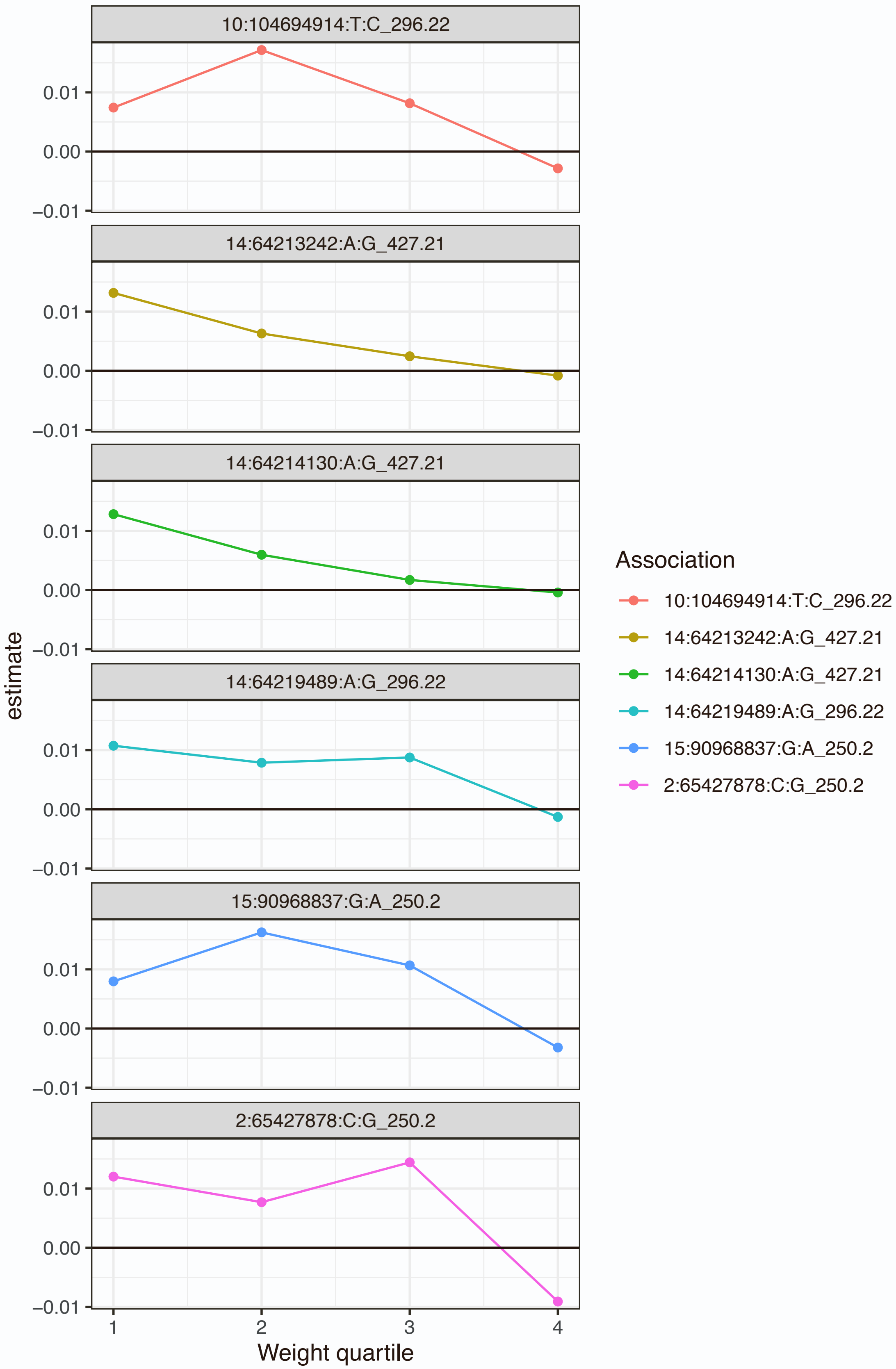

Figure S9. Quartile-stratified effect sizes in associations significant and discordant in the weighted model setting but replicated in the unweighted setting: Unweighted model effect sizes stratified by quartiles of the weight distribution in the 6 associations replicated in the unweighted model setting that were significant with discordant directions of effect in the weighted setting. Model formulations were identical to the aggregate analysis, but run within each individual weight quartile.

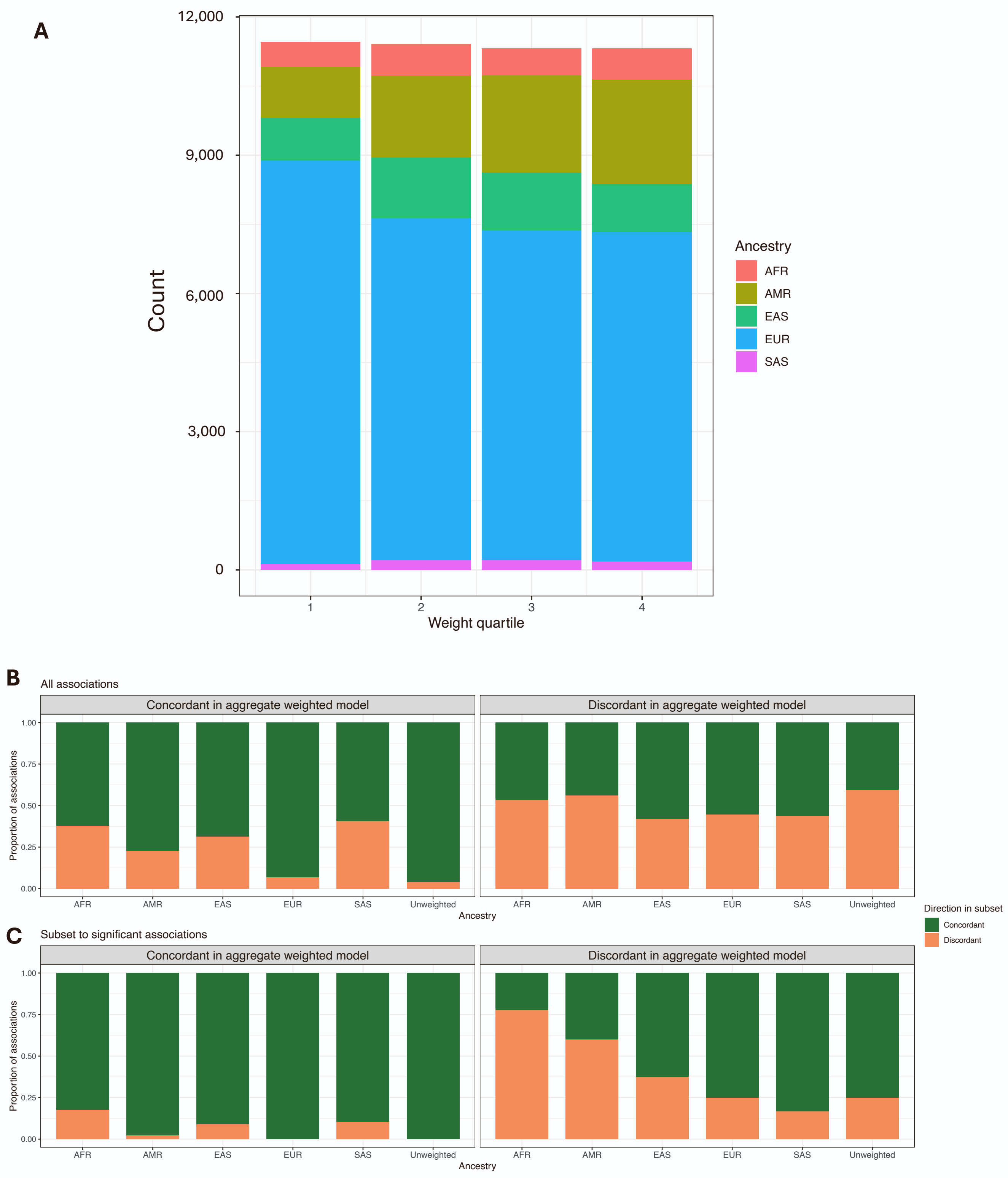

Figure S10. Relationship between genetic ancestry, weight quartile, and concordance of associations: (A) Distribution of genetic ancestry groups across weight quartiles. Within each quartile, individuals for whom an ancestry assignment could not reliably be made were omitted. (B) Proportion of concordant effects of associations in the weighted setting in ancestry-stratified analyses, split by whether the association was significant & concordant (left panel n=724) or significant & discordant (right panel, n=114) compared to pgrm . (C) As in panel B, but restricting associations to those significant in the ancestry-stratified analyses.

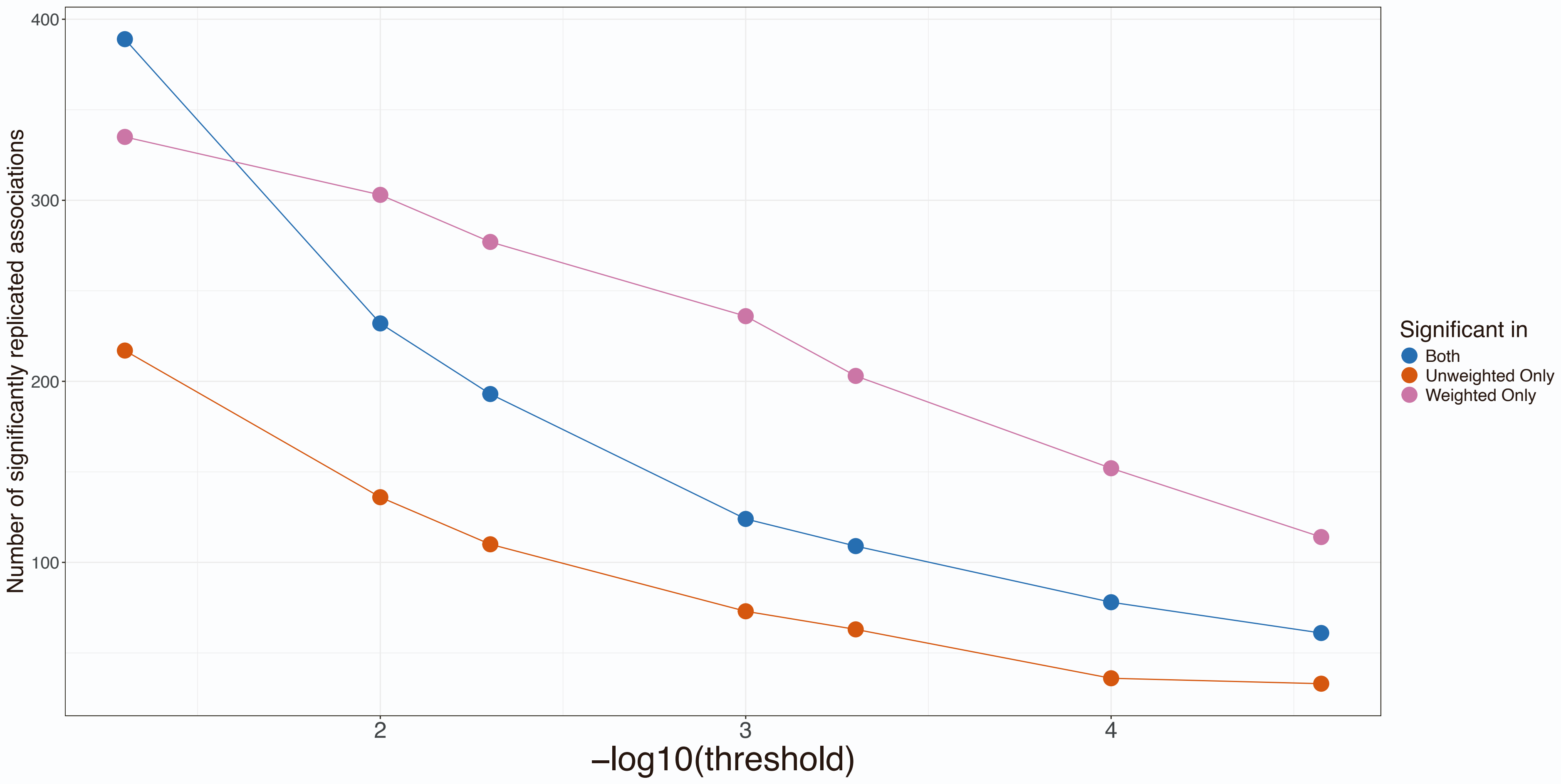

Figure S11. Pgrm variant replication statistics at varying p-value thresholds: Number of significantly replicated associations under each model scheme across p-value thresholds. Replication criteria were p < threshold and consistent direction of effect with pgrm reference.

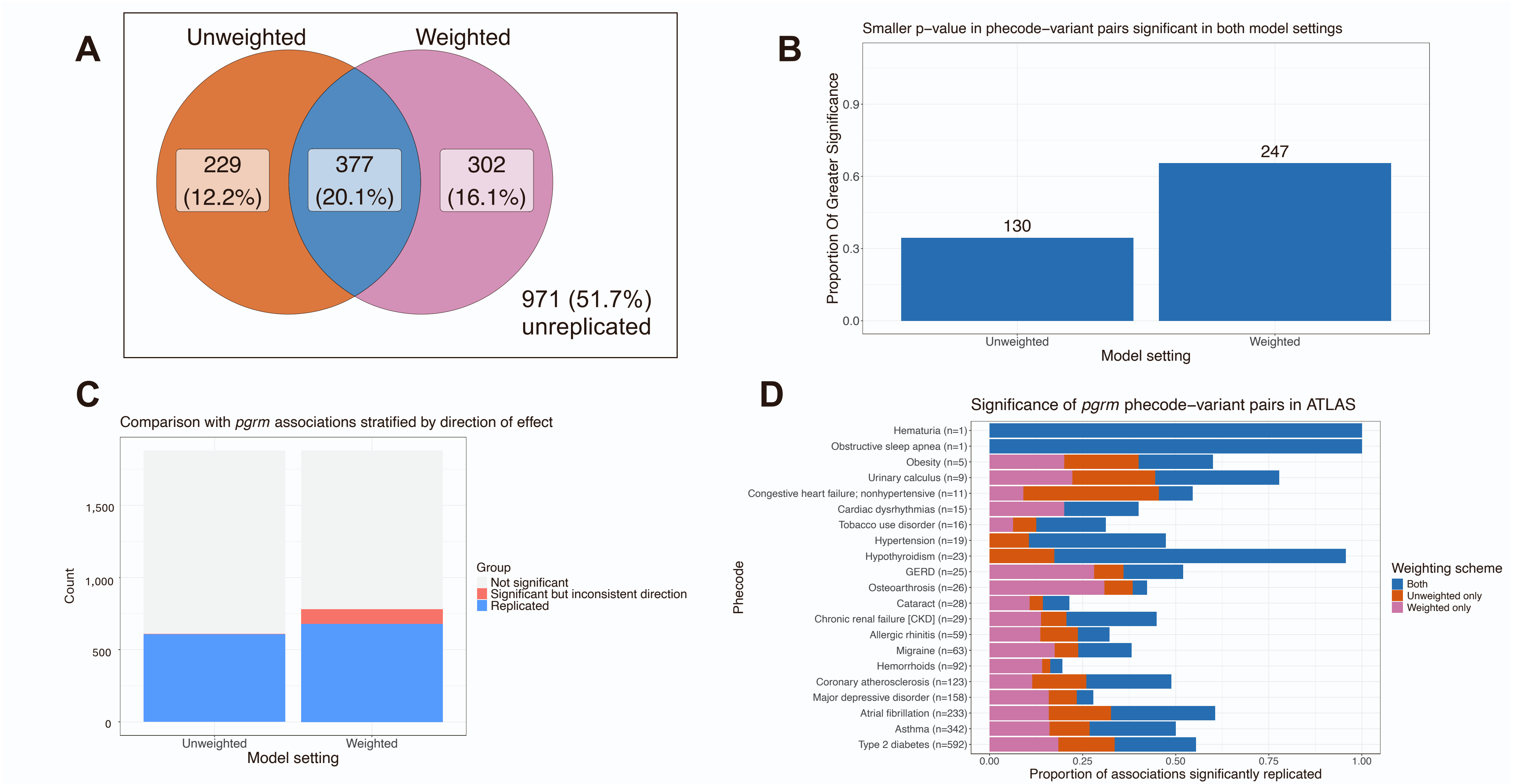

Figure S12. Replication metrics of pgrm variant associations stratified by model setting using the full RF model: Weighted models were adjusted using probabilities from the full random-forest model in place of the recursive-feature eliminated model. A) Venn diagram of counts and percentages of replicated associations from pgrm in the ATLAS sample. Associations were considered replicated at a significance level of $p < 0.05$ and when direction of effect was consistent with pgrm. B) Proportions of associations with smaller p-value stratified by model setting among associations significant under both model settings. C) Proportion of associations with discordant sign with pgrm stratified by model setting. D) Replication across model schemes for variant-phecode associations across 21 phecodes. The number in parentheses indicates the number of queried associations per phecode.

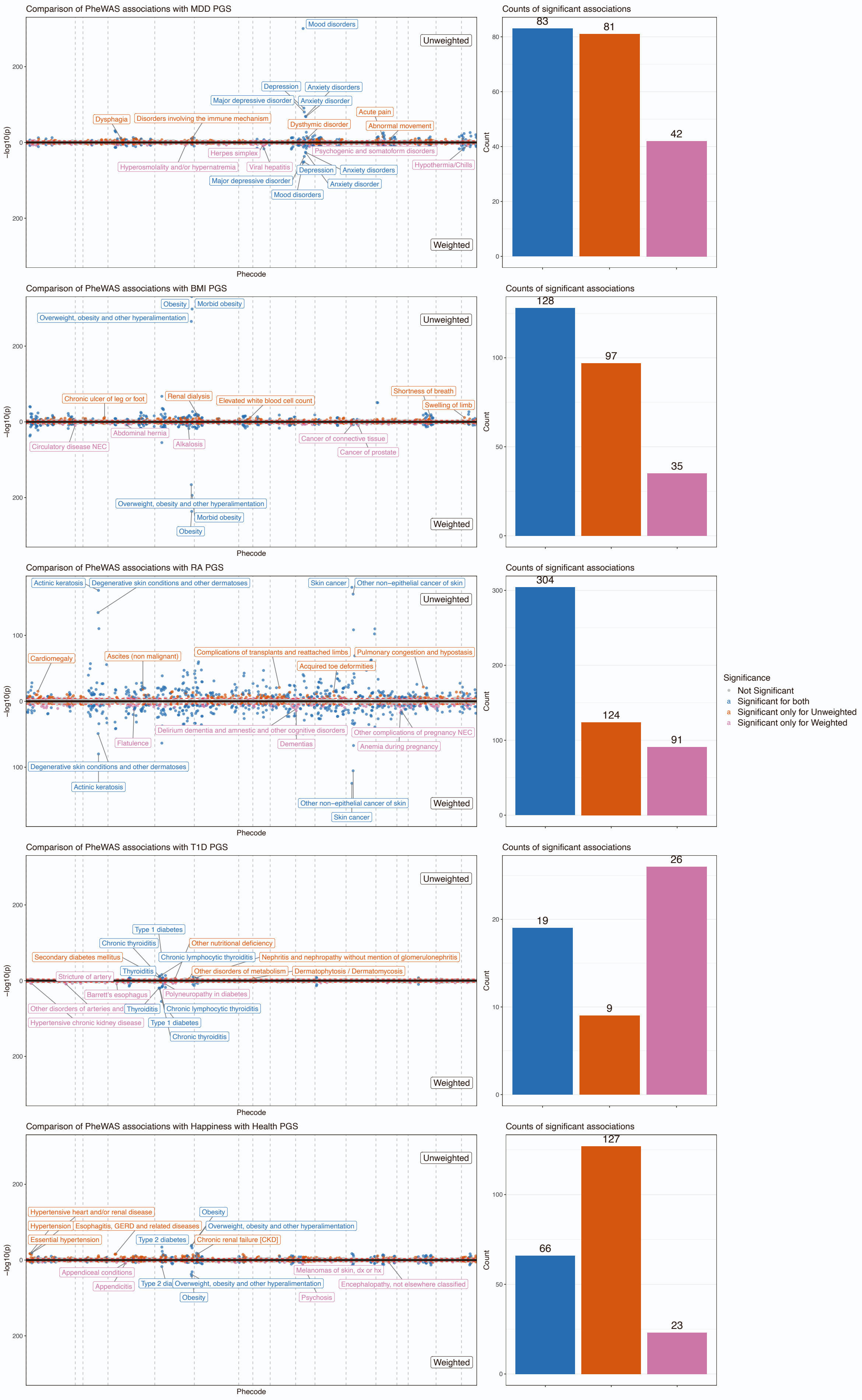

Figure S13. Miami plots of unweighted and weighted PGS-PheWAS associations for 5 PGS: Miami plots for PheWAS on PGS for MDD, BMI, RA, T1D, and Happiness with one's own health (top: unweighted, bottom: weighted) (left). Overall counts of unique and shared associations under model schemes (right)

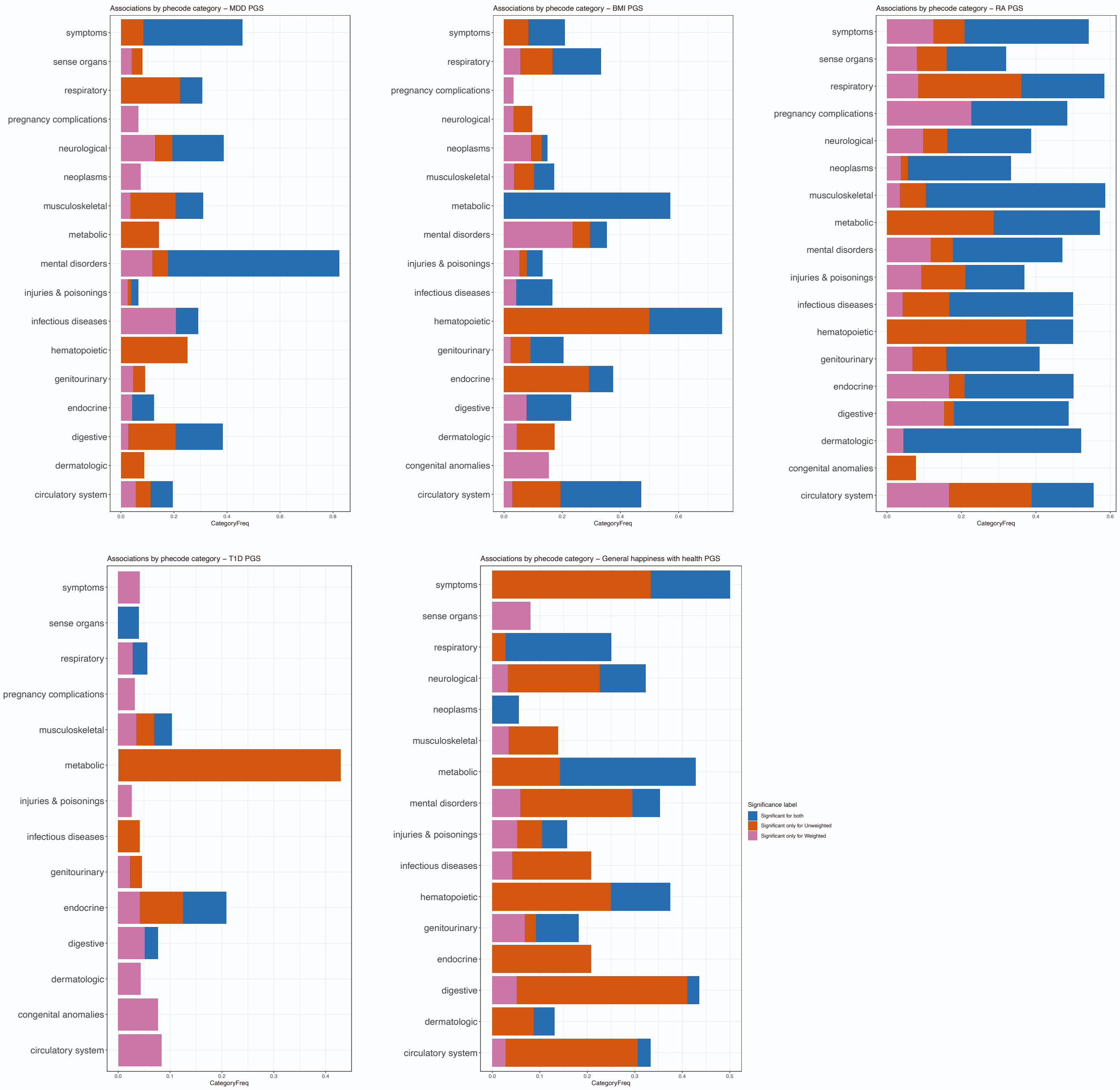

Figure S14. Phecode category breakdown of PheWAS, including associations significant in weighted setting only: Phecode-stratified counts of associations under the unweighted and weighted model schemes, including associations found to be significant only in the weighted setting, for MDD, BMI, RA, T1D, and Happiness with one's own health PGS. Significance was assessed using a Bonferroni-corrected p-value threshold for 1,117 phecodes tested of p < 4.44e-5.

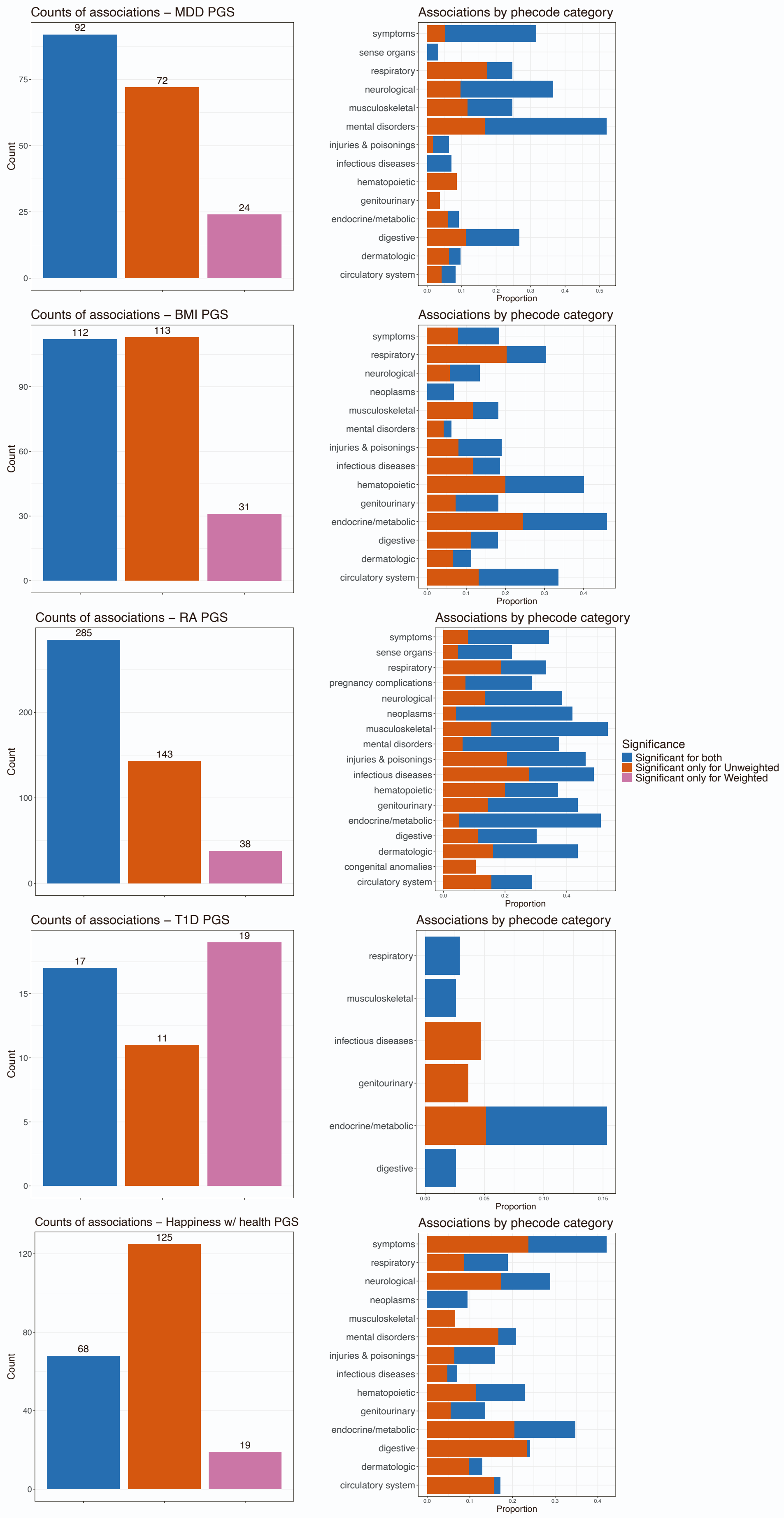

Figure S15. Comparison of unweighted and weighted PGS-PheWAS associations for 5 PGS using the full RF model: Weighted models were adjusted using probabilities from full random-forest model in place of recursive-feature eliminated model. Overall (left) and phecode-stratified (right) counts of shared and unique associations under the unweighted and weighted model schemes for MDD, BMI, RA, T1D, and Happiness with one's own health PGS. Significance was assessed using a Bonferroni-corrected p-value threshold for 1,117 phecodes tested of $p < 4.44e\text{-}5$.

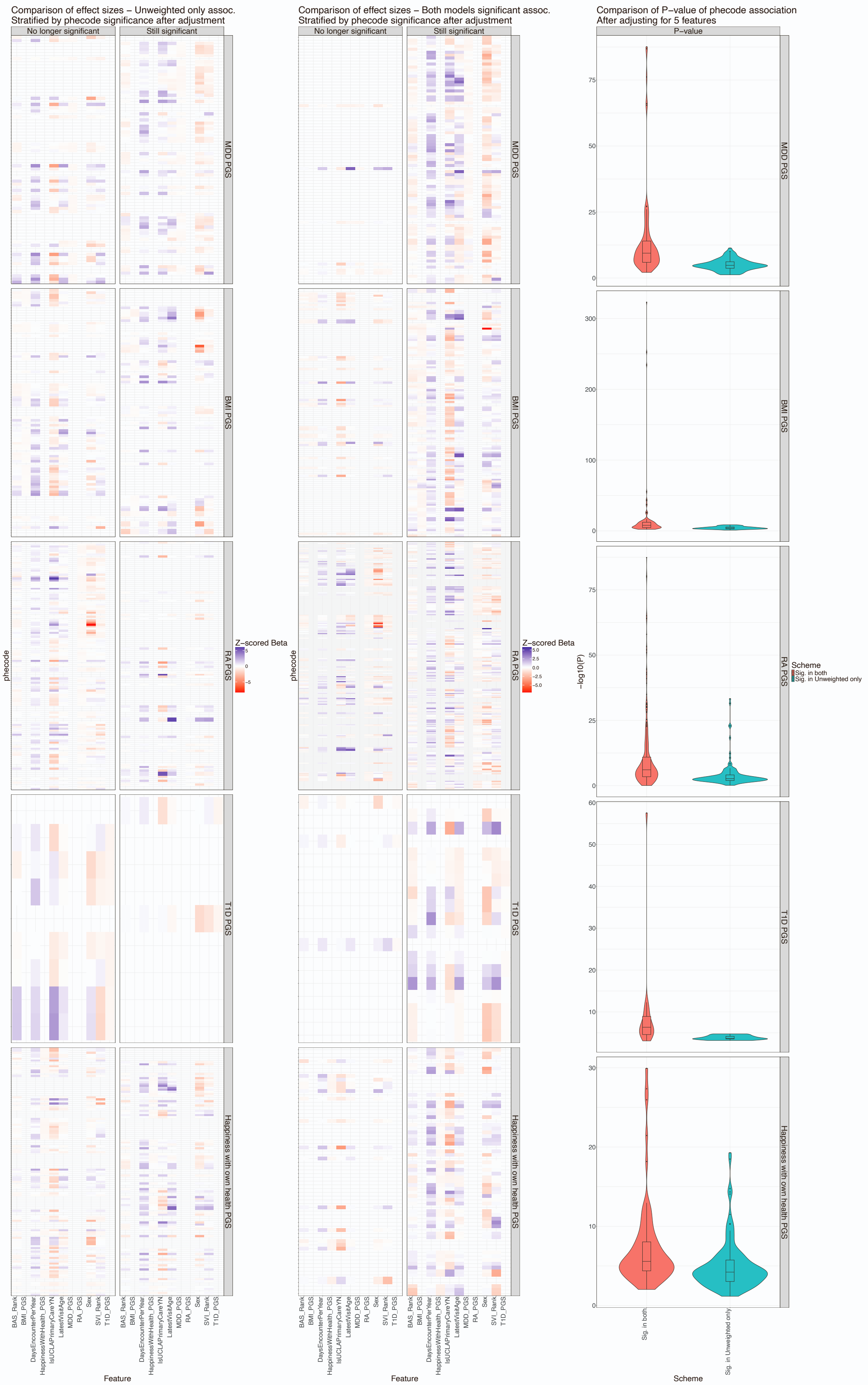

Figure S16. Associations of RF model features following direct adjustment in PGS-PheWAS models: Comparisons of effect sizes (z-scored betas within PGS groups) for RFE random-forest model features after explicit adjustment in PGS-PheAWS models. Comparison in PGS-phecode association settings found to be significant in unweighted models alone (left) and shared by both unweighted and weighted models (middle) stratified by whether phecode-level associations remain significant after accounting for RF model features in PGS-PheWAS models. Comparison of p-values of association with phecode in PGS-PheWAS models accounting for RF model features stratified by phecodes associated with PGS in the unweighted setting alone (red) and in both unweighted and weighted model settings (blue).
